# Adipose gene expression profiles reveal insights into the adaptation of northern Eurasian semi-domestic reindeer (*Rangifer tarandus*)

**DOI:** 10.1038/s42003-021-02703-z

**Published:** 2021-10-07

**Authors:** Melak Weldenegodguad, Kisun Pokharel, Laura Niiranen, Päivi Soppela, Innokentyi Ammosov, Mervi Honkatukia, Heli Lindeberg, Jaana Peippo, Tiina Reilas, Nuccio Mazzullo, Kari A. Mäkelä, Tommi Nyman, Arja Tervahauta, Karl-Heinz Herzig, Florian Stammler, Juha Kantanen

**Affiliations:** 1 Natural Resources Institute Finland (Luke), Jokioinen, Finland; 2 Department of Environmental and Biological Sciences, University of Eastern Finland, Kuopio, Finland; 3 Research Unit of Biomedicine, Faculty of Medicine, University of Oulu, Oulu, Finland; 4 Arctic Centre, University of Lapland, Rovaniemi, Finland; 5 Laboratory of Reindeer Husbandry and Traditional Industries, Yakut Scientific Research Institute of Agriculture, Yakutsk, The Sakha Republic (Yakutia) Russia; 6 NordGen—Nordic Genetic Resource Center, Ås, Norway; 7 Natural Resources Institute Finland (Luke), Maaninka, Finland; 8 Department of Ecosystems in the Barents Region, Norwegian Institute of Bioeconomy Research, Svanvik, Norway; 9 Medical Research Center, Faculty of Medicine, University of Oulu, Oulu, Finland; 10 Oulu University Hospital, Oulu, Finland; 11 Institute of Pediatrics, Poznań University of Medical Sciences, Poznań, Poland

**Keywords:** Gene expression, Fat metabolism

## Abstract

Reindeer (*Rangifer tarandus*) are semi-domesticated animals adapted to the challenging conditions of northern Eurasia. Adipose tissues play a crucial role in northern animals by altering gene expression in their tissues to regulate energy homoeostasis and thermogenic activity. Here, we perform transcriptome profiling by RNA sequencing of adipose tissues from three different anatomical depots: metacarpal (bone marrow), perirenal, and prescapular fat in Finnish and Even reindeer (in Sakha) during spring and winter. A total of 16,212 genes are expressed in our data. Gene expression profiles in metacarpal tissue are distinct from perirenal and prescapular adipose tissues. Notably, metacarpal adipose tissue appears to have a significant role in the regulation of the energy metabolism of reindeer in spring when their nutritional condition is poor after winter. During spring, genes associated with the immune system are upregulated in the perirenal and prescapular adipose tissue. Blood and tissue parameters reflecting general physiological and metabolic status show less seasonal variation in Even reindeer than in Finnish reindeer. This study identifies candidate genes potentially involved in immune response, fat deposition, and energy metabolism and provides new information on the mechanisms by which reindeer adapt to harsh arctic conditions.

## Abstract (web summary)

Weldenegodguad et al. performed transcriptome profiling of adipose tissues from the bone marrow, perirenal, and prescapular fat in Finnish and Even reindeer (in Sakha) during spring and winter. They identified candidate genes potentially involved in immune responses, fat deposition, and energy metabolism that provide insight into the mechanisms by which reindeer adapt to harsh arctic conditions.

## Introduction

Native to northern and subarctic regions of Eurasia, reindeer (*Rangifer tarandus*) have societal, cultural, and ecological values for the livelihoods of arctic indigenous people and pastoralists and have multiple socio-economic roles, such as providing meat, hides, milk, and serving as a means of transportation^1–3^. Reindeer survive in challenging northern and extreme arctic environments characterized by low temperatures, prolonged daylight during summers, and darkness and limited availability of grazing resources during long winters^3,4^. Adipose tissues are vital for reindeer to adapt to such extreme conditions^5,6^. Adipose tissues are important organs for several functions in energy metabolism that are crucial for survival and successful reproduction. White adipose tissue (WAT) stores energy in the form of lipids and serves as a long-term energy reserve, whereas brown adipose tissue (BAT) contributes to both thermohomoeostasis and energy balance by producing heat via the function of uncoupling protein 1 (*UCP1*)^7^. WAT is also an important endocrine organ that secretes several hormones, including adipomyokines and cytokines, which contribute to energy metabolism and immunity and act as signals to the central nervous system^8,9^. The reindeer is a lean animal, but it also relies on WAT as a source of energy and hormonal signals in changing environmental conditions^5^. In newborn reindeer, BAT plays a crucial role in regulating non-shivering thermogenesis, but its effect diminishes over time^10,11^. However, browning of WAT has been observed in various species during later stages of life after chronic cold exposure^12^ and due to pharmacological and nutritional agents^13^. Bone marrow has a specific type of adipose tissue (BMAT) that acts as an energy reservoir, contributes to local and systemic metabolic processes^14,15^, and undergoes dynamic changes^16^. BMAT decreases as a result of starvation^17–19^.

The majority of adipose transcriptome studies using high-throughput RNA sequencing (RNA-seq) have been limited to mice^20,21^, sheep^22–24^, pigs^25–27^, and humans^28–30^. Adipose transcriptomes are affected by the type of adipose tissue, as well as by the sex and age of the animal^31^. Changes in gene expression within adipose tissues in response to temperature fluctuations have been studied mostly in mice so far^20,21,32^. However, gene expression profiles in reindeer adipose tissues have thus far not been investigated. Adipose tissues from various parts of the body have their own unique functions. In the present study, we investigated gene expression profiles of three adipose tissue depots: metacarpal (M), perirenal (P), and prescapular (S) tissues. These adipose depots were selected because they represent visceral (P), peripheral (S), and bone marrow (M) fat. These anatomical depots are also expected to reflect different metabolic functions. For example, the prescapular area is a major BAT depot in newborn reindeer^11^ and thus an interesting target for regulating the expression of *UCP1*, as well as other markers for cold adaptation. The samples were collected from the Finnish and Even (Sakha Republic, Yakutia, Russia) reindeer breeds (or populations), which differ in present-day management and feeding practices. In Yakutia, reindeer feed on natural pastures with extreme seasonal variation and high migratory behaviour (high lichen content in winter, fresh leaves in spring, grass, and mountain herbs in summer), whereas in Finland, extra fodder (concentrates) is provided during peak winter (February and March). Reindeer herders observe that the lichen-rich diet in winter helps reindeer keep their weight and survive the cold, while the grassy diet in summer accounts for the principal annual gain in weight. In Yakutia, reindeer have to survive in extreme climatic conditions where the annual temperature fluctuates from −60 °C up to >+30 °C.

Here we aim to obtain insights into the seasonal fluctuations in the transcriptome profiles of three adipose tissues (metacarpal, perirenal, and prescapular) in two reindeer populations (Even and Finnish). The tissue sampling was conducted during (early) winter (November–December) and (early) spring (April), when the animals were in the best and worst nutritional conditions, respectively. Moreover, to assess the health and physiological condition of the reindeer in different seasons and geographical locations and to complement genetic analyses with phenotypic data, we analysed several blood metabolites along with hormones regulating energy metabolism, such as blood insulin, leptin, and hormone-sensitive lipase (HSL), and proteins such as *UCP1* and *COX4* from adipose tissue. We expect that assessing changes in gene expression in reindeer adipose tissue due to seasonal variation may reveal adaptation mechanisms and help to understand the evolution of this adaptive response in reindeer and other mammalian species sharing northern Eurasian habitat conditions.

## Results

### RNA-seq and mapping

A total of 175 gigabases (Gb) of RNA-seq data were generated from 47 adipose tissue samples collected from 16 Finnish and Even male reindeer. As the adaptors were trimmed automatically, and the Phred quality scores of the reads from all samples were >30, we did not perform further trimming and quality filtering. The number of reads per sample ranged from 26.6 million (M) (4.0 Gb) to 217 M (32.6 Gb), with a mean of high-quality 43.5 M, 2 × 75 bp pair-ended reads per sample (Supplementary Data S1). As shown in Supplementary Data S1, the two samples (FR12_SCAP and YR1_SCAP), revealing the highest numbers of reads (217 M and 165 M, respectively), will be used to detect long non-coding RNAs (lncRNAs) in a future study. The proportion of reads mapped to the reindeer reference genome ranged from 81 to 92%, with, on average, >90% of the reads from each sample uniquely mapped to the reindeer draft genome assembly (Supplementary Data S2). Raw sequence reads in compressed fastq format (fastq.gz) analysed in this study have been deposited to the European Nucleotide Archive and are publicly available under project accession PRJEB44094.

### Gene expression overview

**Fig. 1:**
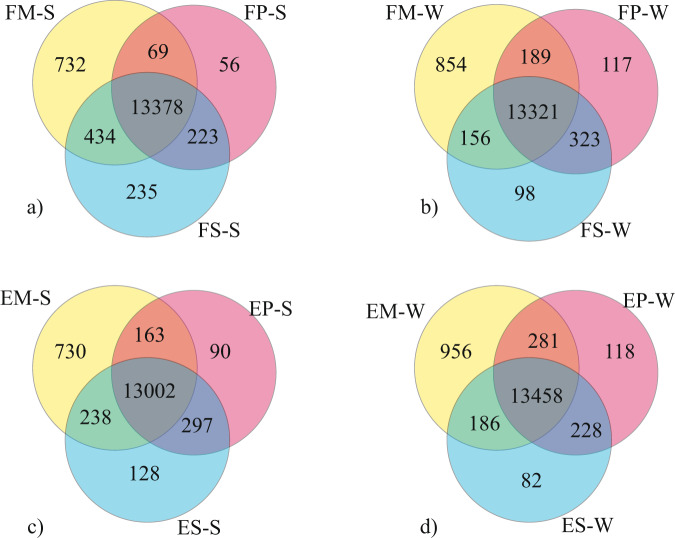
Venn diagram showing overlap of expressed genes (CPM ≥ 0.5 for at least two samples) among tissues in each region and season. Shared and uniquely expressed genes across tissues (metacarpal M, perirenal P, and prescapular S) in **a** Finnish reindeer (F) in the spring (-S), **b** Finnish reindeer (F) in the winter (-W), **c** Even reindeer (E) in the spring (-S), and **d** Even reindeer (E) in the winter (-W).

**Fig. 2:**
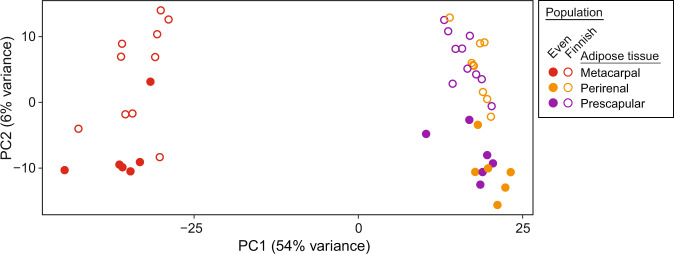
PCA plots of the analysed samples based on expression profiles, with dot colours indicating tissue and region (see legend). The adipose tissue samples are labelled with red (metacarpal), yellow (perirenal), and violet (prescapular) colours. The solid dots represent the Even reindeer samples and the circles the Finnish samples.

A total of 16,212 genes were expressed (count per million (CPM) > 0.5 in at least two samples) in all 47 samples (Supplementary Data S3), representing approximately 60% of the 27,332 reindeer genes reported in the draft reindeer genome assembly annotation file^3^. The highest number of genes were expressed in metacarpal adipose tissue (*n* = 15,554), followed by prescapular (*n* = 14,998) and perirenal (*n* = 14,837) adipose tissues. Moreover, we examined the expressed genes across these three tissues to search for shared and uniquely expressed genes in the respective region and season (Fig. 1a–d). In all the cohorts (spring and winter Finnish and Even samples), the highest number of expressed genes was found in the metacarpal adipose tissue. This tissue also displayed a higher number of tissue-specific expressed genes than did the perirenal and prescapular adipose tissues (Fig. 1a–d). In various region–season comparisons between the adipose tissues, >12,800 genes were commonly expressed. To assess expression similarity among the samples, we performed principal component analysis (PCA) based on the top 500 most variable genes (Fig. 2 and Supplementary Fig. S1a–c). The PCA plot (Fig. 2) shows that the metacarpal tissue samples were clustered separately from the other two tissues, and additional grouping of the samples along axis 2 based on two reindeer breeds. Similarly, a hierarchal clustering based on the top 25 genes with the highest variance across all samples also showed a similar pattern that clearly separated metacarpal tissue and the other tissues (Supplementary Fig. S2).

**Fig. 3:**
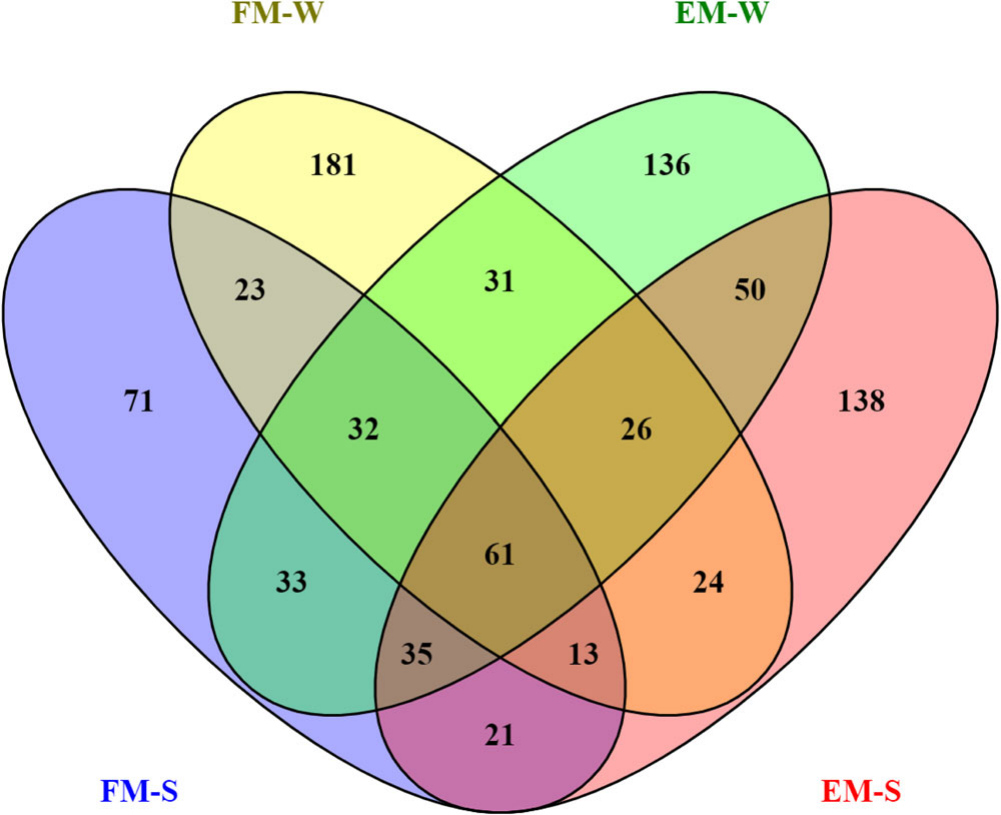
Distribution of uniquely expressed genes in the metacarpal adipose tissue (M). Shared and uniquely expressed genes in Finnish (FM-W, FM-S) and Even (EM-W, EM-S) reindeer in the spring (-S) and winter (-W) are shown in the Venn diagram.

While >12,800 genes were commonly expressed in all tissues, several tissue-specific genes were identified in this study (Supplementary Data S4). Moreover, 30 of the differentially expressed genes (DEGs) were common in 3 tissues (Supplementary Fig. S3). In line with the distinct cluster observed in the PCA plot, >730 genes were uniquely expressed in the metacarpal adipose tissue (Fig. 1). We further explored the genes specific to this tissue by removing the lowly expressed genes (transcripts per million < 1). Out of the 875 genes, 61 were commonly expressed in metacarpal adipose tissue in the different experimental groups. The metacarpal adipose tissue of Finnish reindeer collected in the winter had the largest number of uniquely expressed genes (*n* = 181), whereas Finnish metacarpal tissue collected in the spring possessed the lowest (*n* = 71) number of uniquely expressed genes. By contrast, Even reindeer samples collected in either season had roughly similar numbers of uniquely expressed genes (138 in spring and 136 in winter samples; Fig. 3).

Among the 61 genes that were unique to metacarpal adipose tissue yet shared by Finnish and Even reindeer, annotations were not available for 10 genes. The metacarpal-specific genes included several homeobox proteins (e.g. *HOXD13*, *HOXA11*, *HOXD11*, *HOXA13*, *DLXC*), bone sialoprotein 2 (*IBSP*), osteomodulin (*OMD*), carbonic anhydrase 3 (*CA3*), C-X-C motif chemokine 10 (*CXCL10*), nuclear receptor-interacting protein 3 (*NRIP3*), and R-spondin-2 (*RSPO2*) (Supplementary Data S5).

### Seasonal differences in gene expression

For an insight into the effects of seasonal conditions, we compared gene expression profiles of adipose tissues from spring vs. winter samples separately in the Finnish and Even reindeer. Finnish reindeer showed a higher number of significant DEGs than the Even reindeer (Table 1).

**Table 1:** The number of significantly identified differentially expressed genes (DEGs) in Finnish (F) and Even (E) reindeer due to seasonal changes (S, spring; W, winter) in the metacarpal (M), perirenal (P), and prescapular (S) adipose tissues.

Comparison	Total DEGs	Upregulated	Downregulated
FM-S vs. FM-W	346	195	151
FP-S vs. FP-W	583	273	310
FS-S vs. FS-W	611	325	286
EM-S vs. EM-W	156	57	99
EP-S vs. EP-W	103	45	58
ES-S vs. ES-W	176	126	50

Altogether 346 genes were differentially expressed between seasons in metacarpal tissues of Finnish reindeer; of these, 195 were upregulated in spring samples and the rest were upregulated in the winter samples (Table 1, Supplementary Data S6, and Supplementary Fig. S4). Genes involved in metabolism (*ANGPTL8*, *BDH1*, *ASIP*, *QPRT*) and stress response (*BOLA3*, *GPR158*) were strongly upregulated in spring (log_2_ fold change (LFC) > 4) and those particularly associated with immune functions (*CCL11*, *CXCL8*, *CD33*, *IL1B*, *IRF4*) were strongly downregulated (LFC < −3.8) in spring metacarpal tissue (Supplementary Data S6 and Supplementary Fig. S4).

Among 583 DEGs in the perirenal tissue, genes such as *CCL11*, *TRH*, *MT1A*, *RPL38*, *AREG*, *NPW*, *SRSF3*, *RPS29*, *RPL36A*, and *SLC11A1*, were highly upregulated (LFC > 4) in the spring samples and *MP68*, *RPL39*, *SLC39A12*, *SCN3A*, *RGS9*, *HSPA6*, *TNFRSF10B*, *EDIL3*, *RTP1*, and *ABCC4* were highly downregulated (LFC < −3.7) (Table 1, Supplementary Data S7, and Supplementary Fig. S5). The DEGs upregulated in the spring samples include genes participating in the immune system (*SLC11A1*, *COMMD6*, *BATF*, *CCL19*) and ribosomal and transcription processes (*RPL38*, *RPL36A*, *FKBP11*). On the other hand, downregulated genes were associated with signalling or signal transduction (*RGS9*, *RAPGEF5*, *COL4A5*,* PTGER2*, *MAML3*, *PDE5A*), cell differentiation or organogenesis (*GJA5*, *THSD7A*, *DACH1*, *MMRN2*, *NHSL2*, *ZFHX3*), and interaction of glucose and fatty acid metabolism (PDK4, SIK1).

The highest number (*n* = 611) of DEGs was found in prescapular tissue, 325 of which were upregulated in the spring samples (Table 1, Supplementary Data S8, and Supplementary Fig. S6). In prescapular adipose tissue from the spring, genes such as *SERPINA3-7*, *ESD*, *APOH*, *SERPINI2*, *SERPINC1*, *TCEB2*, *ITIH1*, *WNT9B*, *C8B*, *TRH*, *ASCL2*, *GSTA2*, and *VPREB1* were among the top upregulated genes (LFC > 4.5) and *LHFP*, *TNFRSF10B*, *CRISP3*, *NEGR1*, *KCNA2*, *PTGER2*, *HSPA6*, *GTSF1*, and *RPL30* were among the top downregulated genes (LFC < −3.5) (Supplementary Data S8 and Supplementary Fig. S6). A total of 30 genes, including *APOLD1*, *CYP26B1*, *IGFBP5*, *MICA*, *MICB*, *MT-ATP8*, *PDK4*, and *SCP2*, were commonly differentially expressed in three tissues (Supplementary Fig. S3 and Supplementary Data S9). Many of the genes in the prescapular tissue that were upregulated during spring were associated with inflammatory or immunological responses (*FXYD5*, *CCL19*, *IFIT3*, *CARD9*, *GMFG*, *SLC11A1*), feeding behaviour (*NPW*), adipogenesis (*DLK1*, *CITED4*), cell growth or differentiation (*MGP*, *ECSCR*, *TIMP1*, *MT1A*), and spermatogenesis (*SCHBP1L*, *MORN2*). Similarly, downregulated DEGs were associated with signalling (*RGS5*, *DCBLD2*, *RASGEF1B*, *RAPGEF5*), organogenesis (*PTGER2*, *KLF7*, *PHACTR2*, *KMT2A*, *DACH1*, *PDLIM5*), and lipid metabolism (*ARFGEF3*, *PDK4*, *FOXO1*, *PITPNC1*).

Similar pairwise comparison in Even reindeer revealed 156, 103, and 176 DEGs in metacarpal, perirenal, and prescapular tissues, respectively (Table 1, Supplementary Data S10–S12, and Supplementary Figs. S8–S10). Prescapular adipose tissue had the highest number of unique significant DEGs (*n* = 141), followed by metacarpal (*n* = 135) and perirenal (*n* = 68) (Supplementary Fig. S7). In both breeds, prescapular tissue harboured the highest (611 in Finnish and 176 in Even reindeer) number of DEGs (Supplementary Data S8 and S12). Interestingly, while the number of upregulated genes was higher in the metacarpal tissue of Finnish samples from the winter collection, the same was not observed in Even reindeer. In the Even reindeer, we found seven common DEGs (*ORC5*, *ADI1*, *RPS15A*, *DAGLA*, *JCHAIN*, *ENSP00000353290*, and *FEZ1*) among all tissues (Supplementary Fig. S7). DEGs that were upregulated in the spring samples of Even metacarpal adipose tissue appeared to have roles in lipid/fatty acid metabolism (*ELOVL7*, *APOA1*, *ACOT2*, *ANGPTL1*), cell structural functions (*CAPN6*, *MYL9*, *CLDN5*), and oxygen metabolism (*FMO1*, *FMO2*, *AOC1*, *STEAP1*). Downregulated DEGs in Even metacarpal adipose tissue were associated with development and organogenesis (*DMAP1*, *COL27A1*, *CA2*, *NRG3*, *JAK3*, *EPB41L3*, *ESM1*) and immune system (*C4A*, *IL17RB*, *HLA-DOA*, *SIGLEC1*, *CPA3*). In Even perirenal adipose tissue, several immunoglobulin-related genes were upregulated during spring, suggesting activation of the immune system by pathogens. However, downregulated genes were mainly associated with lipid/energy metabolism (*ACACB*, *PRLR*, *APOL6*) and functions related to growth and development (*MEGF8*, *DAGLA*, *IGSF10*, *GRIP2*, *NSMF*). Among the three adipose tissues in Even reindeer, immune-related DEGs (e.g. *JCHAIN*, *IGHM*, *IGKC*, *IGHV3-6*, *IGLC7*, *MUCM*) were predominantly upregulated in the prescapular tissue, whereas downregulated genes in the prescapular tissue were associated with lipid/energy metabolism (*TP53INP2*, *PPARGC1B*, *ACACB*) and growth and development (*DAGLA*, *IGS10*, *BMP5*, *FGFR2*, *NRG3*, *CYP26B1*).

### Gene expression differences between the Finnish and Even reindeer

We made six pairwise comparisons (three each for spring and winter samples) to identify DEGs between Finnish and Even reindeer (Table 2 and Supplementary Data S13–S18) of which the highest number (*n* = 504) of DEGs was present in metacarpal tissues collected during winter, whereas the same tissue revealed the lowest number (*n* = 126) of DEGs during spring (Table 1 and Supplementary Data S13 and S16). In comparisons involving spring samples, the highest numbers of DEGs were found in prescapular adipose tissue.

**Table 2:** The number of significantly identified DEGs in the metacarpal (M), perirenal (P), and prescapular (S) adipose tissues of Finnish (F) and Even (E) reindeer that were compared separately for spring (-S) and winter (-W) samples.

Comparison	Total DEGs	Upregulated	Downregulated
EM-S vs. FM-S	126	78	48
EP-S vs. FP-S	301	189	112
ES-S vs. FS-S	385	229	156
EM-W vs. FM-W	504	336	168
EP-W vs. FP-W	401	182	219
ES-W vs. FS-W	365	154	221

Four genes, *FIBP*, *CREB3L3*, *CLDN4*, and *ALKBH3*, were exclusively upregulated in the Finnish reindeer irrespective of the seasons. *FIBP* (*FGF1 Intracellular Binding Protein*) is known to promote mitogenic action to induce morphogenesis and differentiation. *CREB3L3* has been linked to triglyceride metabolism and growth suppression. *CLDN4* (*Claudin 4*) is a member of the claudin gene family. Being integral membrane proteins, claudins, in general, have a vital role in regulating the transport of solutes and ions through calcium-independent cell-adhesion activity.

Similarly, *TMEM182*, *AACS*, *FAM159B*, and *C19ORF80* were always upregulated in all five comparisons except between EM-S vs. FM-S. Among the four genes, we did not find any relevant information about the function of *FAM159B*. There is relatively little information available on *TMEM182*, but its upregulation may be associated with adipose growth and remodelling^33,34^. In the present context, a greater abundance of *TMEM182* in Even reindeer males might be linked to castration, as castrated males are known to accumulate more adipose tissues. *AACS* (*Acetoacetyl-CoA Synthetase*) appears to be involved in ketone body metabolism during adipose tissue development. *C19ORF80* (alternatively *ANGPTL8*, *Angiopoietin-like 8*) is known to mediate the transition between fasting and re-feeding and has an important role in the storage of fatty acids in adipose tissue during the refeeding state^35^. Interestingly, *C19ORF80* was downregulated in Even reindeer compared to Finnish reindeer among spring samples. We speculate that the additional winter feeding for the Finnish reindeer might have a role in the differential expression of *C19ORF80*. Moreover, several of the DEGs were shared between pairwise comparisons (Supplementary Fig. S11 and Supplementary Fig. S12).

### Enriched GO terms associated with DEGs resulting from seasonal comparisons: Finnish reindeer

Enrichment analyses based on significantly DEGs revealed several Gene Ontology (GO) terms and Kyoto Encyclopaedia of Genes and Genomes (KEGG) pathways, thus highlighting the important biological and physiological activities in each tissue. From the 346 significant DEGs between FM-S and FM-W (Table 1 and Supplementary Data S6), 112 genes lacked GO annotations. Separate GO enrichment analyses were performed for the downregulated (*n* = 117 with GO annotation) and upregulated (*n* = 117 with GO annotation) genes. GO analysis indicated that 16 and 36 GO terms were significantly associated with downregulated and upregulated DEGs, respectively (Supplementary Data S19 and Supplementary Fig. S13). Downregulated DEGs were represented in GO terms mainly associated with immune processes (e.g. “immune system process”, “immune response”) and stimulus (e.g. “response to stimulus”, “response to chemical”, “chemotaxis”) (Supplementary Fig. S13a), whereas upregulated DEGs were represented in terms associated with metabolic processes (“ATP metabolic process”, “nucleoside metabolic process”, “single-organism metabolic process”, “carbohydrate derivative metabolic process”), and transport (e.g. “proton transport”, “hydrogen transport”, “cation transmembrane transport”) in the metacarpal adipose tissue of Finnish reindeer (Supplementary Fig. S13b).

In contrast to metacarpal adipose tissue, both the perirenal and prescapular adipose tissues had downregulated genes represented in a relatively higher number of GO terms (*n* = 29, *n* = 55, respectively; Supplementary Data S20 and Supplementary Fig. S14) than upregulated genes (*n* = 16, *n* = 7, respectively; Supplementary Data S21 and Supplementary Fig. S15). The DEGs upregulated during spring in Finnish reindeer perirenal adipose tissue were associated with metabolic (“cellular amide metabolic process”, “organonitrogen compound metabolic process”) and biosynthetic (“amide biosynthetic process”, “organonitrogen compound biosynthetic process”) processes (Supplementary Fig. S14a) and those upregulated during winter were associated with signalling (“signalling”, “G-protein coupled receptor signalling process”, cell communication”, “single organism signalling”, “signal transduction”) and regulation (“regulation of biological process”, “regulation of cellular process”, “biological regulation”) related biological processes (Supplementary Fig. S14b). Altogether, 35 of the 55 GO terms associated with DEGs upregulated in the prescapular adipose tissue of Finnish reindeer from winter sampling were categorized under “biological process” (Supplementary Fig. S15a), whereas none of the seven GO terms enriched in upregulated DEGs of spring samples were categorized under “biological process” (Supplementary Fig. S15b). Moreover, GO terms associated with the regulation of several processes (e.g. “regulation of metabolic process”, “regulation of RNA biosynthetic process”, “regulation of biological process”, “regulation of gene expression”), response (e.g. “cellular response to organic cyclic compound”, “response to steroid hormone”, “response to lipid”, “response to hormone”, “cellular response to stimulus), and transcription activities (e.g. “transcription coactivator activity” and “transcription cofactor activity”) categorized upregulated DEGs of Finnish prescapular adipose tissue collected during winter (Supplementary Fig. S15a).

### Enriched GO terms associated with DEGs resulting from seasonal comparisons: Even reindeer

The differential expression analysis between EM-S and EM-W revealed 156 significantly DEGs (Table 1 and Supplementary Data S10), of which 55 lacked GO annotations. GO enrichment analysis of the downregulated genes (*n* = 77 with GO annotations) yielded 7 significantly represented GO terms (Supplementary Data S22 and Supplementary Fig. S16), including “metalloendopeptidase activity” and “metallopeptidase activity”, whereas no significantly enriched GO terms were found in the upregulated genes. In Even reindeer perirenal tissue, of the 103 significantly DEGs in EP-S vs. EP-W (Table 1 and Supplementary Data S11), 32 genes did not have GO annotations. Gene enrichment analysis of the upregulated and downregulated DEGs revealed no statistically significantly represented GO terms. From the list of 176 significantly DEGs between ES-S and ES-W (Table 1 and Supplementary Data S12), 59 genes lacked GO annotation. Gene enrichment analysis of the upregulated and downregulated DEGs revealed no statistically significantly represented GO terms.

### Enriched GO terms associated with DEGs resulting from location comparison

DEGs resulting from the comparison of the metacarpal adipose tissue between Even and Finnish reindeer did not reveal any GO terms. Upregulated genes in the perirenal adipose tissue of Even reindeer from spring sampling were associated only with the GO term “cofactor binding”, whereas downregulated genes were not enriched in any GO terms. Similar comparison of perirenal adipose tissue from winter sampling revealed 8 and 46 GO terms associated with upregulated and downregulated genes, respectively, in Even reindeer (Supplementary Data S23).

In spring prescapular adipose tissues of Even reindeer, a total of 19 GO terms including signalling (e.g. “single organism signalling”, “signal transduction”, “G-protein coupled receptor signalling pathway”) and response to stimulus (“response to external stimulus”, “cellular response to stimulus”) were associated with upregulated genes (Supplementary Data S24), while none of the downregulated genes were enriched in any GO terms. Similar comparison of winter samples in Even reindeer revealed 3 overrepresented GO terms (“anion binding”, “cofactor binding” and “pyridoxal phosphate binding”) and 20 underrepresented GO terms (including 6 signalling terms, “G-protein coupled receptor activity”, “receptor activity” and “cell communication”) (Supplementary Data S25).

### KEGG pathways associated with seasonal comparisons: Finnish reindeer

We performed KEGG pathway analysis using GAGE to identify pathways that were differentially regulated by season (FM-S vs. FM-W). Pathway enrichment analysis revealed 18 and 5 significantly downregulated and upregulated KEGG pathways, respectively, in spring metacarpal adipose tissue (Supplementary Data S26). Most of the downregulated pathways were associated with the immune system, environmental information processing, and signal transduction (Supplementary Data S26). Pathways associated with immune system included “cytokine-cytokine receptor interaction”, “chemokine signalling pathway”, “Fc gamma R-mediated phagocytosis”, “IL-17 signalling pathway”, and “T cell receptor signalling pathway” (Supplementary Data S26). In addition, the significantly downregulated pathways in spring metacarpal tissue associated with environmental information processing and signal transduction included: “TNF signalling pathway”, “MAPK signalling pathway”, “NF-kappa B signalling pathway”, “Jak-STAT signalling pathway”, and “ErbB signalling pathway” (Supplementary Data S26). Similarly, the significantly upregulated pathways in spring metacarpal tissue included “ribosome”, “oxidative phosphorylation”, “biosynthesis of secondary metabolites”, “microbial metabolism in diverse environments”, and “biosynthesis of antibiotics” (Supplementary Data S26).

KEGG pathway analysis using DEGs of FP-S vs. FP-W comparison indicated that 12 and 3 pathways were significantly downregulated and upregulated in spring perirenal tissue, respectively (Supplementary Data S27). The downregulated pathways were mainly associated with environmental information processing and signal transduction, such as “cAMP signalling pathway”, “cGMP-PKG signalling pathway”, “Rap1 signalling pathway”, “Hippo signalling pathway”, and “MAPK signalling pathway” (Supplementary Data S27). However, pathways such as “ribosome”, “oxidative phosphorylation”, and “ribosome biogenesis in eukaryotes” (Supplementary Data S27) were significantly upregulated in spring perirenal tissue.

KEGG pathway analysis for FS-S vs. FS-W DEGs revealed two and nine significantly upregulated and downregulated KEGG pathways in spring prescapular tissue, respectively (Supplementary Data S28). Pathway analysis showed a significant upregulation of two pathways, “ribosome” and “complement and coagulation cascades”. The downregulated pathways were mainly associated with environmental information processing and signal transduction: “Hippo signalling pathway–fly”, “cAMP signalling pathway”, “ErbB signalling pathway”, “MAPK signalling pathway”, “Hippo signalling pathway”, and “FoxO signalling pathway”.

### KEGG pathways associated with seasonal comparisons: Even reindeer

Pathway analysis in Even reindeer revealed three KEGG pathways significantly upregulated in the metacarpal adipose tissues collected during spring (“ribosome”, “spliceosome”, and “ribosome biogenesis in eukaryotes”), whereas no significantly downregulated pathways were found. Analysis of DEGs from perirenal adipose tissue did not reveal any KEGG pathways. Pathways such as “complement and coagulation cascades” and “cytokine–cytokine receptor interaction” were significantly upregulated during spring in prescapular adipose tissue, while no KEGG pathways were associated with downregulated genes.

### KEGG pathways associated with location comparisons

DEGs from the comparison of the metacarpal adipose tissue between Even and Finnish reindeer did not reveal any KEGG pathways associated with upregulated genes, whereas two KEGG pathways, “oxidative phosphorylation” and “ribosome”, were associated with genes downregulated in Even reindeer in spring (Supplementary Data S29) and a similar comparison from winter sampling revealed seven KEGG pathways, such as “ribosome biogenesis in eukaryotes”, “TNF signalling pathway”, “IL-17 signalling pathway”, “NF-kappa B signalling pathway”, and “cytokine-cytokine receptor interaction” associated with downregulated genes (Supplementary Data S30).

Upregulated genes in the perirenal adipose tissue of Even reindeer from spring sampling were associated with one KEGG pathway “ribosome”, while downregulated DEGs were not associated with any of the pathways. Similar comparison from winter sampling revealed 6 and 13 KEGG pathways associated with upregulated and downregulated genes, respectively, in Even reindeer (Supplementary Data S31).

While no KEGG pathways were associated with upregulated genes in prescapular adipose tissues from spring samples, two (“ribosome” and “complement and coagulation cascades”) were linked to the downregulated genes. Similar comparison in winter samples revealed 14 and 1 KEGG pathways associated with upregulated and downregulated genes, respectively, in Even reindeer (Supplementary Data S32).

### Immunoblotting of UCP1 and COX4

**Fig. 4:**
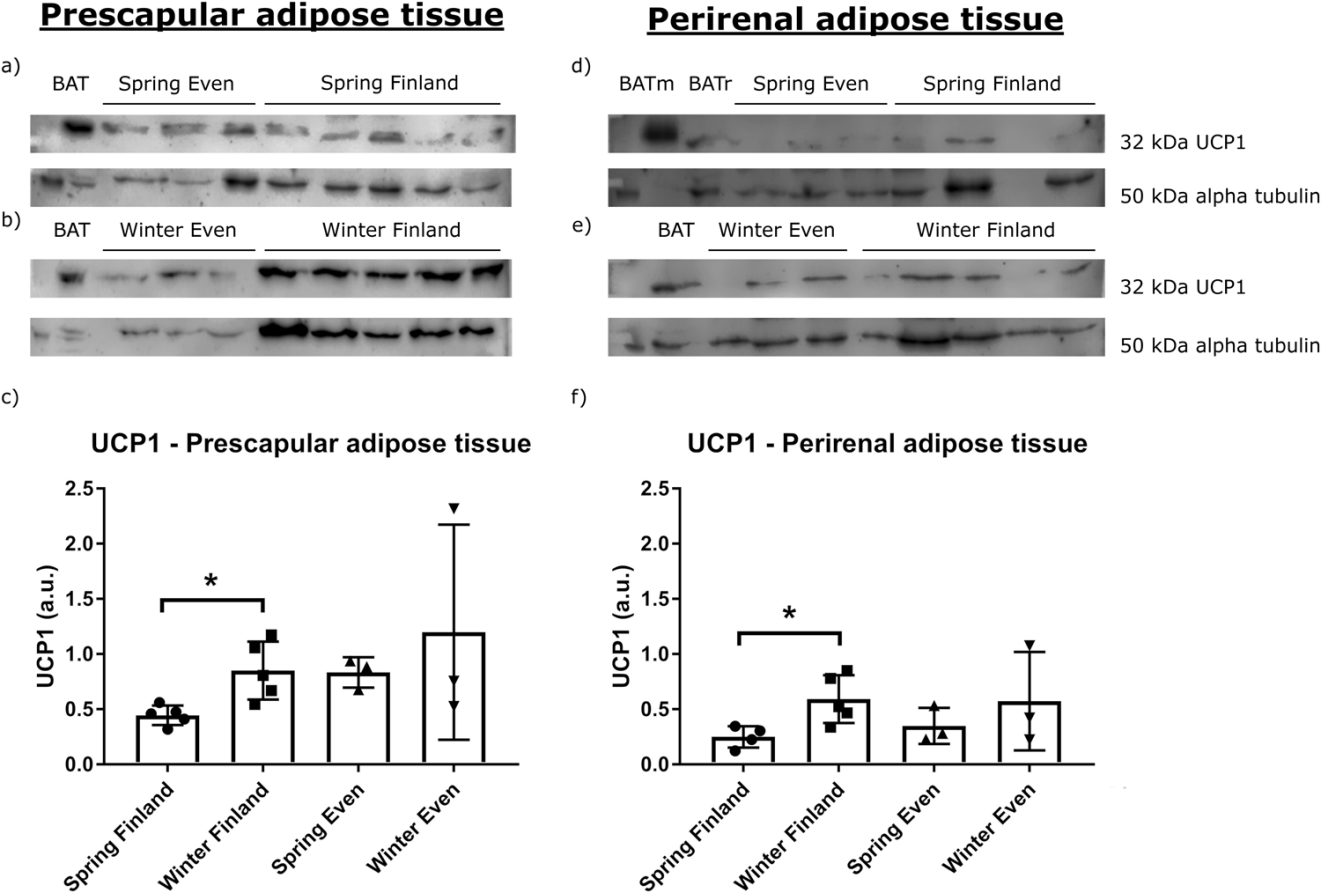
UCP1 expression in each seasons and regions. The western blots and relative expression of UCP1 from **a**–**c** reindeer prescapular and **d**–**f** perirenal adipose tissue total proteins. The upper blots on the left side show UCP1 content in prescapular adipose tissue **a** in spring in Even reindeer (*n* = 3) and Finnish reindeer (*n* = 5), **b** in winter in Even reindeer (*n* = 3) and Finnish reindeer (*n* = 5), and the lower graph shows **c** their relative expression levels. The upper blots on the right side show UCP1 content in perirenal adipose tissue **d** in spring in Even reindeer (*n* = 3) and Finnish reindeer (*n* = 4), **e** in winter in Even reindeer (*n* = 3) and Finnish reindeer (*n* = 5), and the lower graph shows **f** their relative expression levels. Samples contained 50 µg of total protein per lane, except the reindeer BAT (brown adipose tissue; BAT and BATr) samples with 5 µg and mouse BAT (BATm) with 1 µg of protein. Alpha tubulin was used as a loading control. The relative expression of UCP1 was normalized to alpha tubulin and presented as mean arbitrary units (a.u.) ± SD (**c**, **f**). Significant differences between seasons are indicated with a bar and an asterisk (*p < 0.05).

Immunoblotting on UCP1, a protein specific to thermogenic adipocytes, was conducted on cross-section of different adipose tissues. Immunoreactivity at 32 kDa molecular weight characteristic of UCP1 was evident, although in small amounts, in the total proteins of prescapular and perirenal adipose tissues of both Even and Finnish reindeer (Fig. 4a, b, d, e). In general, prescapular adipose tissues appeared to have slightly higher UCP1 expression than perirenal adipose tissues. In Finnish reindeer, the relative expression of UCP1 was significantly higher in winter compared with spring in both prescapular (*p* = 0.0016*) and perirenal (*p* = 0.032*) adipose tissues (Fig. 4c, f). The Even reindeer exhibited a similar albeit not statistically significant trend of higher UCP1 expression in winter compared with that in spring (Fig. 4c, f).

**Fig. 5:**
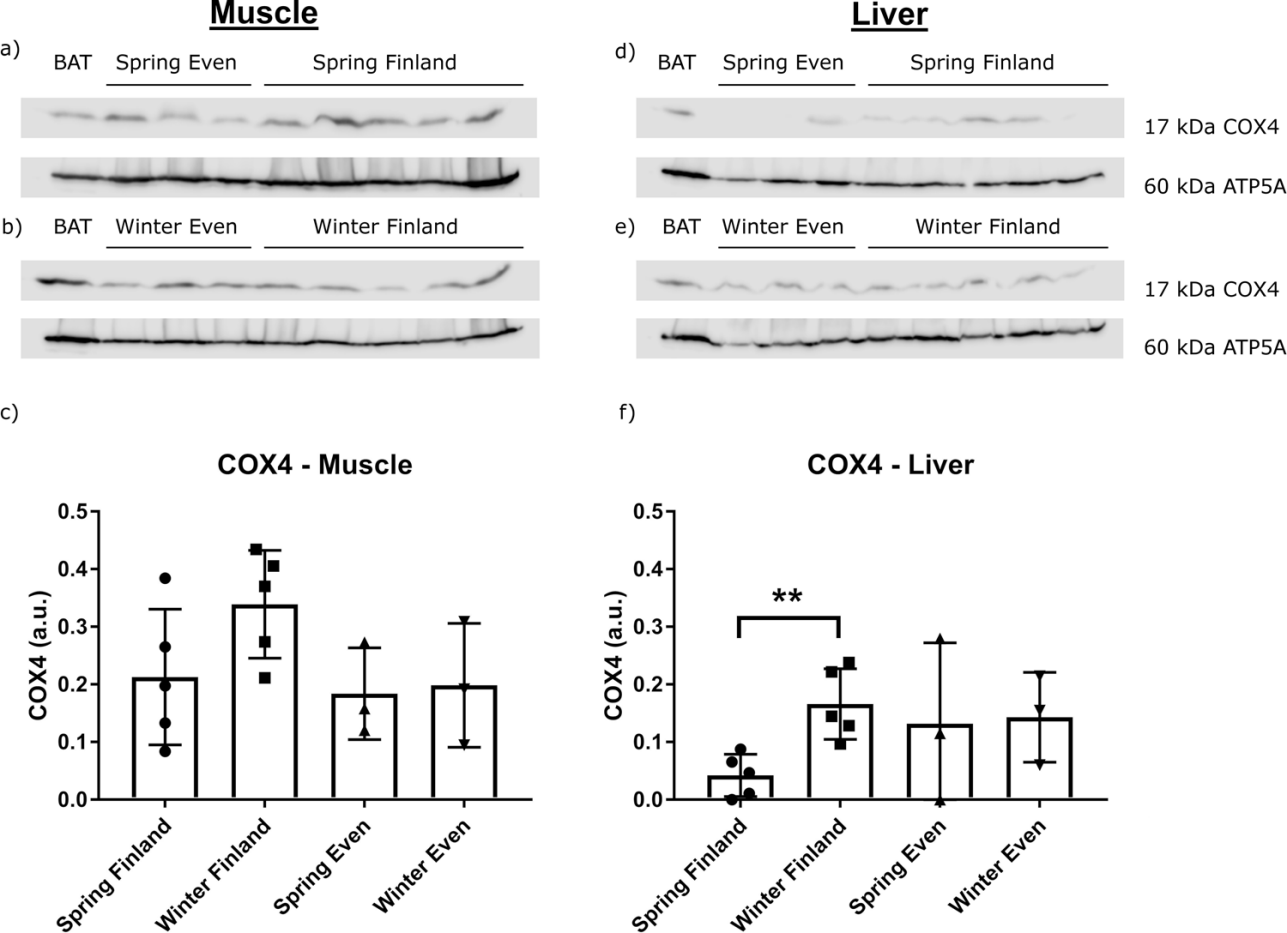
COX4 expression in each seasons and regions. The western blots and relative expression of *COX4* from **a**–**c** reindeer muscle (*M. gluteobiceps femoris*) and **d**–**f** liver mitochondrial proteins. The upper blots on the left side show COX4 content in the muscle **a** in spring in Even reindeer (*n* = 3) and Finnish reindeer (*n* = 5), **b** in winter in Even reindeer (*n* = 3) and Finnish reindeer (*n* = 5), and the lower graph shows **c** their relative expression levels. The upper blots on the right side show *COX4* in the liver **d** in spring in Even reindeer (*n* = 3) and Finnish reindeer (*n* = 5), **e** in winter in Even reindeer (*n* = 3) and Finnish reindeer (*n* = 5), and the lower graph shows **f** their relative expression levels. Samples contained 10 µg of mitochondrial protein per lane, except the reindeer BAT (brown adipose tissue) sample with 1 µg of mitochondrial protein. Mitochondrial ATP synthase (ATP5A) was used as a loading control. The relative expression of *COX4* was normalized to *ATP5A* and presented as mean arbitrary units (a.u.) ± SD (**c**, **f**). Significant difference between seasons is indicated with a bar and asterisks (**p ≤ 0.01).

The expression of COX4, an enzyme central to oxidative phosphorylation, was undetectable from the adipose tissue total proteins. However, we analysed its expression in other metabolically active tissues—the liver and muscle (see “Methods”; Fig. 5a, b, d, e). The relative expression level of COX4 in the liver samples was significantly higher in Finnish reindeer in winter (*p* = 0.008**) in comparison to spring (Fig. 5c, f). There were no statistically significant differences in muscle COX4 levels between seasons.

### Blood metabolites

**Fig. 6:**
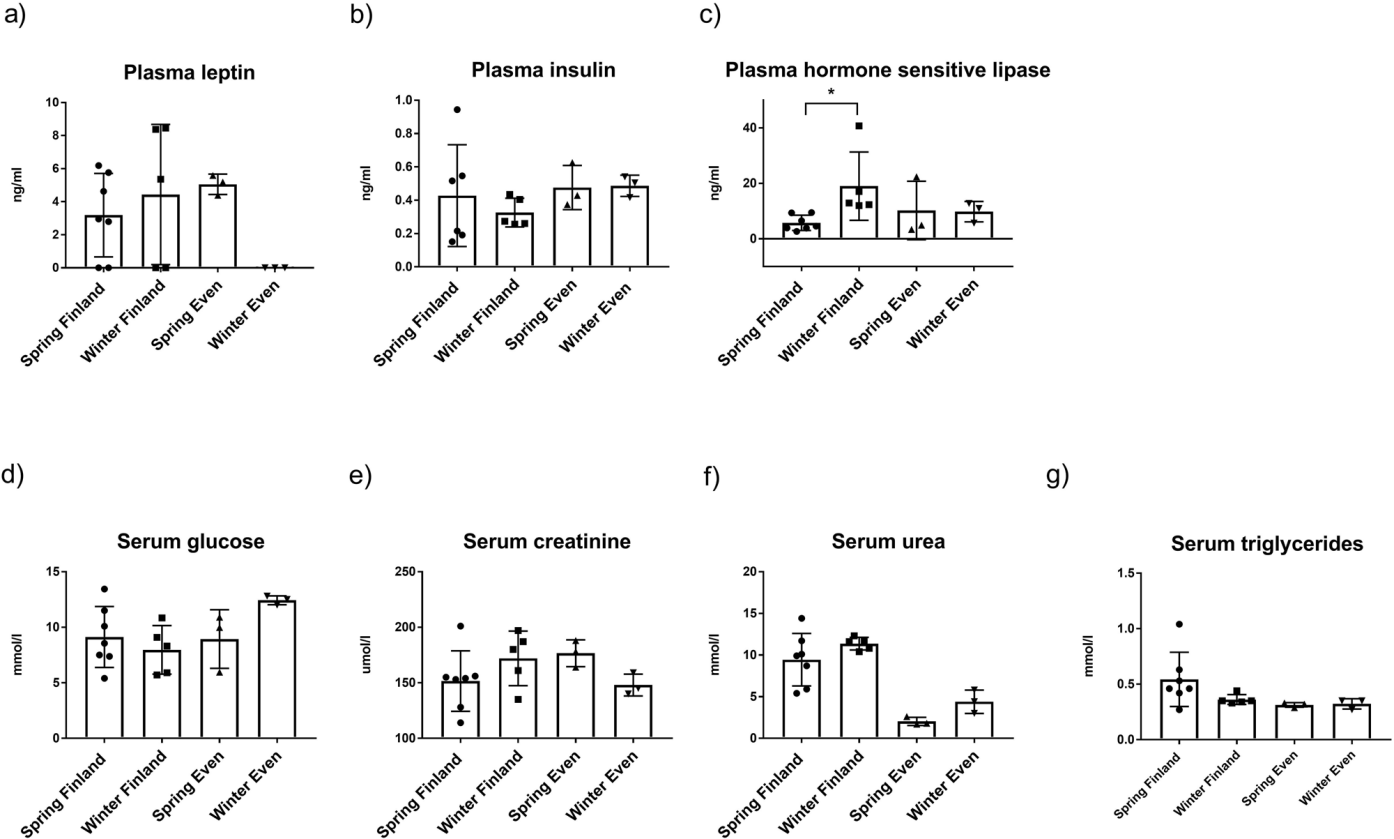
Plasma hormone and serum metabolite levels in Finnish male reindeer in winter (*n* = 5) and spring (*n* = 7) and in male Even reindeer in winter (*n* = 3) and spring (*n* = 3). Plasma hormones **a** leptin, **b** insulin, and **c** hormone-sensitive lipase (HSL) and serum metabolites **d** glucose, **e** creatinine, **f** urea, and **g** triglyceride levels are presented as mean ± SD. Significant difference between seasons is indicated with a bar and an asterisk (*p ≤ 0.01).

Plasma leptin levels were at a similar range in Even and Finnish reindeer in spring but under the detection limit (1.56 ng/ml) in Even reindeer in winter (Fig. 6a). Plasma insulin levels of reindeer were at similar level in both seasons and regions (Fig. 6b). Plasma HSL was significantly higher in winter in Finnish reindeer compared with that in spring (*p* = 0.033*), but there were no significant differences in the Even reindeer between seasons (Fig. 6c).

Serum glucose, creatinine, and triglyceride concentrations were at a similar range in both Even and Finnish reindeer in both seasons (Fig. 6d, e, g). There was a trend of higher serum urea (Fig. 6f) in winter samples compared to spring samples within the reindeer of both regions, albeit not statistically significant.

## Discussion

Adipose tissues are vital for animals living in cold environments, promoting adaptation by temperature regulation, energy homoeostasis, regulation of fat deposition, and metabolism^5,6,10,11,36–40^. Here we have investigated gene expression profiles of adipose tissues from three different anatomical depots in Finnish and Even reindeer during two seasonal time points. To the best of our knowledge, this is the first transcriptome study of adipose tissues in reindeer. Our results indicated a clear difference in gene expression profiles in metacarpal adipose tissue compared to perirenal and prescapular adipose tissues. We found that, during the less optimal circumstances in early spring, mainly characterized by undernutrition, genes associated with the immune system were upregulated in perirenal and prescapular adipose tissues, while genes involved in energy metabolism were upregulated in metacarpal tissue. Interestingly, developmental, growth, and adipogenesis processes were downregulated in all three tissues during spring.

RNA-seq is an efficient method to screen new genes, transcripts, gene expression, and DEGs in various organisms, tissues, and cells^41–43^. In this study, we identified a total of 16,212 expressed genes in focal adipose tissues, which appeared to cover approximately 60% of the list of genes available for the reindeer reference genome^3^. The present results revealed an adequate number of expressed genes in reindeer adipose tissues, which were then utilized in subsequent gene expression analysis to investigate genes associated with seasonal variation (early spring vs. early winter) and location (Even reindeer vs. Finnish reindeer).

In this study, highly abundant genes were associated with fat and lipid metabolism, thermogenesis, and energy homoeostasis that are critical for the survival of reindeer during seasonal fluctuations. Several of the abundant genes involved in fat and lipid metabolism, such as *FABP4*, *FABP5*, *MT-CYB*, and *ADIPOQ* (Supplementary Data S33), were highly expressed in all adipose tissues in both Finnish and Even reindeer. *Fatty acid binding protein 4* (*FABP4*) was the most highly expressed gene in all adipose tissues in both Finnish and Even reindeer. Three of the top expressed genes, including *FABP4*, *FABP5*, and *ADIPOQ*, were also found to be highly expressed in a previous adipose transcriptome profiling study in two fat-tailed sheep breeds^22^. Moreover, *FABP4* and *FABP5* were also highly expressed in the perirenal tissue of sheep^44^. *FABPs 4* and *5*, encode the FABP found in adipocytes and epidermal cells, respectively. A previous study showed that *FABP4* and *FABP5* play an important role in thermogenesis during cold exposure and starvation^45^. Moreover, previous studies in cattle have suggested that *FABP4* plays a crucial role in fat deposition, fatty acid transport, catabolism, and metabolism^22,46,47^. Adiponectin (*ADIPOQ*), secreted by adipocytes and exclusively expressed in adipose tissues, plays an important role in modulating the regulation of fatty acid oxidation, glucose levels, and insulin sensitivity^22,48,49^. The top expressed genes associated with fat metabolism observed in the present study may play a vital role in energy homoeostasis, thermoregulation, and promoting adaptation of reindeer to the challenging environment. The blood lipid, glucose, and insulin levels of reindeer support the view of homoeostasis despite challenging conditions.

Tissue-wise, the gene expression profiles of metacarpal adipose tissue were remarkably different from those of prescapular and perirenal adipose tissues. The highest number of tissue-specific genes was found in metacarpal adipose tissue (Fig. 1). Similarly, the PCA plot based on all expressed genes revealed a cluster for metacarpal adipose tissue, which was distinct from the samples representing other tissues (Fig. 2). We found that several genes from the homeobox (HOX) family of proteins were uniquely expressed in metacarpal adipose tissue. These genes are known to play important roles in the differentiation of adipocytes^50^. Furthermore, several genes associated with cytokines and immune response appeared to be downregulated in the metacarpal tissue during spring (Supplementary Data S6 and S10). By contrast, DEGs associated with cytokines and immune response were upregulated in the perirenal and prescapular tissues during spring (Supplementary Data S7, S8, S11, and S12). Hence, these results indicate the unique biological functions of the metacarpal tissue compared to the other two tissues.

The distinctiveness of the metacarpal adipose tissue may be due to its local niche functions in bone marrow, although it also contributes to systemic metabolism^14,16^. BMAT has many properties in common with WAT, but it is an adipose type of its own that is currently being actively studied^14,16^. In addition to its role as a local energy reservoir and its contribution to haematopoiesis and osteogenesis^15^, metacarpal adipose tissue also secretes a variety of hormones and proteins, such as adiponectin and leptin, which have an important function in the regulation of energy metabolism^16^. The metabolic profile of metacarpal adipose tissue is composed of both white and brown fat, indicating its plasticity in performing different functions^16^.

We observed more seasonal differences in the number of DEGs in the adipose tissues of Finnish reindeer (*n* = 1229) compared to Even reindeer (*n* = 386, also see Table 1). The Finnish reindeer specifically exhibited several significant DEGs associated with ATPase and ATP synthase, whereas no ATP-related genes were detected in Even reindeer. The significant DEGs associated with ATPase and ATP synthase identified in Finnish reindeer include *ATP12A*, *ATP5L*, *ATP5O*, *ATP6AP1L*, and *ATP8* in metacarpal, *ATP1A2*, *ATP1B2*, *ATP6V0C*, *ATP7A*, and *ATP8* in perirenal, and *ATP1A2*, *ATP1B2*, *ATP7A*, *ATP8B1*, *ATP8B3*, and *ATP8* in prescapular adipose tissue. A previous study on zebrafish (*Danio rerio*) reported that feeding altered the expression of ATP-related genes^51^. The differences between Even and Finnish reindeer could be due to the influence of management (extra feeding in Finland vs. no additional feeding in Yakutia), vegetation (relatively sparser in Yakutia), and temperature (relatively warmer in Finland). A further possible explanation for the high expression of ATP genes in Finnish reindeer is that they were fed concentrates with a high protein content (~10% protein content) compared to their natural winter food, lichens (2–3% protein content).

In terms of seasonal comparison, we observed that the gene expression profiles of metacarpal tissue were mainly enriched for energy metabolism instead of their typical role in immune systems. Adipose tissue has been previously reported to play a role in immune and inflammatory systems^52,53^. Earlier studies reported that adipocytes of both peripheral and bone marrow fat secrete a variety of hormones and proteins, such as pro-inflammatory and anti-inflammatory cytokines^54–56^. However, in the present Even reindeer metacarpal tissue, the downregulated genes in spring included genes associated with cytokines, such as *CCL19*, *IGDCC4*, *IL17RB*, *JCHAIN*, *LECT1*, and *IGLV1-51*. Moreover, several of the downregulated DEGs in spring in Finnish and Even reindeer metacarpal adipose tissues revealed genes associated with immune system. On the other hand, the upregulated DEGs in spring in Finnish and Even reindeer metacarpal adipose tissue revealed genes associated with lipid and energy metabolism, supporting the view that bone marrow fat acts as a source of energy when reindeer are in poor condition^18,19^. In reindeer, the proportions of unsaturated fatty acids, oleic, and linoleic acid are significantly decreased in metatarsal bone marrow fat in poor conditions in spring^19^. This may be related to their use for oxidation or synthetic processes. For instance, angiopoietin-like protein 8 (*ANGPTL8*) and angiopoietin-like protein 1 (*ANGPTL1*) were among the upregulated genes detected in the metacarpal tissue of Finnish and Even reindeer, respectively, in spring. *ANGPTL8* is a member of the angiopoietin-like protein (*ANGPTL*) family involved in the metabolic transition from fasting to re-feeding and plays a key role in lipid metabolism^57–59^. Depending on the location in the body, adipose tissues differ in terms of cellular composition, quantity, and proportion of adipocytes and their capacity to produce adipocytokines^9^. During starvation, adipose tissue often limits the cytokine levels to reduce the consumption of resource/energy by the immune systems to actively decrease energy usage; subsequently, the activity of immune cells is limited^60–62^. Reindeer in both locations, and particularly Even reindeer, are in their poorest nutritional condition during spring. In this study, all cytokine genes identified in the metacarpal tissue were downregulated in spring. This might be due to the metacarpal adipose tissue being exclusively involved in thermogenesis and energy metabolism by suppressing the function of the immune system. Hence, when these reindeer experience the worst conditions during spring, the stored fat depots from metacarpal adipose tissues may be exclusively reserved for energy metabolism.

As revealed by the top downregulated genes in the perirenal and prescapular adipose tissues of Finnish reindeer and in all three adipose tissues of Even reindeer, biological processes associated with development, cell growth, and organogenesis were repressed during spring. The downregulation of genes involved in organogenesis, cell growth, and development in spring may indicate that the animals give less priority for growth- and development-related processes during extreme conditions; thus, the animals spend no extra energy for growth-related processes. As mentioned above, during extreme conditions, the animals reduce several metabolic activities to increase the efficiency of energy usage.

We also analysed adipose *UCP1* and *COX4* protein levels as indicators of the potential thermogenesis and metabolic state of the reindeer. We anticipated that adipose tissues of the adult reindeer are WAT but may potentially have some BAT characteristics, considering the extreme long-term exposure of the reindeer to cold, especially in Siberia. There is no previous evidence of BAT or “browning” of adipose tissues in adult reindeer, but it is well established that newborn reindeer have active BAT at birth and during their first month of life^10,11^.

Our results show the presence of *UCP1* protein in two different adipose tissues of adult reindeer, prescapular and perirenal depots (Fig. 4). Relative *UCP1* expression was significantly higher in winter compared with spring in Finnish reindeer in both prescapular and perirenal adipose tissues, likely reflecting colder weather conditions in winter. A similar trend was observed in the Even reindeer. Due to the low amount of protein, it is likely that *UCP1* does not have major thermogenic relevance. However, the findings are still interesting, as they show that *UCP1* was present in the WATs of adult reindeer. They also refer to plasticity of adipose tissue, that is, the potential to adjust its functions according to prevailing conditions^63^. In general, reindeer can cope with very low ambient temperatures in winter (−30 °C) without increasing their heat production^64^, and thus non-shivering thermogenesis is usually not necessary. This is mainly due to the good insulation capacity of the winter coat of reindeer, which effectively prevents heat loss. Liver *COX4* expression was significantly higher in Finnish reindeer in winter as compared to in spring, and a similar trend was apparent in Even reindeer (Fig. 5). This indicates an increase of oxidative phosphorylation and ATP synthesis in the liver to match the increased energy demands caused by the colder season.

Plasma leptin and insulin levels were low (Fig. 6) and agree with earlier findings in reindeer^5^. Leptin is a hormone secreted by adipose tissues and plays a role in the regulation of body weight^65^. The low leptin levels suggest that the adipose tissues of reindeer were small and that the animals were striving to preserve their adipose tissues. Low leptin also agrees with adipose transcriptomics results referring to fat mobilization. Plasma HSL was significantly higher in Finnish reindeer in the early winter than in the spring group, suggesting the mobilization of storage lipids to accommodate increased energy expenditure of male reindeer related to the breeding season.

Serum creatinine, and urea concentrations were similar between seasons (Fig. 6), indicating that the harsh winter season is relatively well tolerated by the reindeer without severe muscle catabolism, which would be indicated by changes in the blood parameters.

Collectively, our mRNA-seq data uncovered variations in the transcriptome profiles of three adipose tissues in relation to seasonality and location. In general, our study showed that highly expressed genes in adipose tissues were associated with fat and lipid metabolism and thermal and energy homoeostasis, promoting the adaptation of reindeer to challenging northern Eurasian environments. Further, our results indicated a distinct gene expression profile in metacarpal adipose tissue compared to perirenal and prescapular adipose tissues. Metacarpal adipose tissue appeared to have a greater role in metabolic activities compared to the other two tissues especially during spring when the animals are experiencing the worst nutritional conditions. Moreover, reindeer from Finland and Yakutia displayed different gene expression profiles, in part owing to climatic and management differences. Thermogenic UCP1 protein was present in adipose tissues of both Even and Finnish reindeer, although in low amounts, showing that the reindeer have an option for extra heat production. Taken together, the results and resources from this study will be useful for elucidating the genetics and physiology of adipose tissue for adaption to northern Eurasian conditions.

## Methods

### Sample collection for transcriptome analysis

This study includes RNA-seq of 47 tissue samples from 16 reindeer males that were randomly collected at slaughter from two different geographical regions (Inari, northern Finland and Eveno-Bytantay, Sakha, Yakutia, the Russian Federation) at two seasonal time points: winter (November–December) and spring (April) (Table 3 and Supplementary Data S1). Perirenal samples were taken from the adipose tissue around the kidneys, prescapular samples from the adipose tissue located beneath the cervical muscles in front of the scapula, and metacarpal samples from the bone marrow in the diaphysis of the metacarpal bone (left front leg). For convenience, throughout the text, the sample groups are abbreviated using reindeer location (F, E), tissue type (P, S, M), and seasonal time points (S, W). For example, FM-S represents the metacarpal tissue of Finnish reindeer collected during spring. The samples were stored in RNAlater® Solution (Ambion/QIAGEN, Valencia, CA, USA). It should be noted that three male reindeer (spring) from Yakutia were castrated, whereas those from Finland (*n* = 10) and autumn Yakutian males (*n* = 3) were uncastrated. The animals grazed on natural pastures throughout the year before the sampling. Reindeer diet on natural pastures consists of the available seasonal plants: fresh green vegetation in summer, autumn, and spring, additionally mushrooms in the autumn and mostly lichen in the winter season. In addition to grazing on natural pastures, the Finnish reindeer were fed concentrates including on average 10 MJ/kg metabolizable energy and 10% protein in dry matter (Poroherkku, Raisio, Finland) for 2–8 weeks in February and March 2016 and kept in feeding pens prior to slaughter. The diet of Finnish reindeer is often supplemented by this type of concentrates by reindeer herders in the critical winter season, as the natural lichen might be sparse, to ensure the wellbeing and survival of the herd. In Siberia with the vast grazing areas, this is not used. The animals were exposed to seasonal ambient temperatures and photoperiod. The mean daily temperature in Inari, Finland varied between −16.1 and 5.2 °C before the sampling in winter (14 h light, 10 h dark) and between −13.2 and 4 °C before the sampling in spring (16 h light, 8 h dark). In northern Sakha, the daily temperature varied between −13 and −24 °C during the winter sampling (6.5 h daylight, 17.5 h dark) and between −9 and 0 °C during the spring sampling (14 h of daylight, 10 h dark). Serum and plasma samples were also collected from Sodankylä, Finland in the spring (15 h light, 9 h dark), where the mean daily temperatures varied between −11.4 and 3.7 °C. All protocols and sample collections were performed in accordance with the legislations approved by the Russian authorization board (FS/UVN 03/163733/07.04.2016) and the Animal Experiment Board in Finland (ESAVI/7034/04.10.07.2015).

**Table 3:** Summary of adipose tissue samples used for RNA-seq and physiological studies (the latter in brackets).

	Finnish reindeer		Even reindeer	
	Spring male	Winter male	Spring male^a^	Winter male
Metacarpal	5	5	3	3
Perirenal	4 (4–5)	5 (5)	3 (3)	3 (3)
Prescapular	5 (5)	5 (5)	3 (3)	3 (3)

Blood samples were collected from all the animals and two additional male Finnish reindeer in spring.

^a^Castrated males (Spring samples).

### Sample collection for physiological analysis

Blood samples were taken before slaughter by a jugular venipuncture into vacuum serum and EDTA K3 tubes. The blood samples were centrifuged, and the separated serum and plasma were stored at −80 °C until analysis. A total of 18 males, Finnish (*n* = 12) and Even (*n* = 6) reindeer, were examined, of which 16 were included in the RNA-seq analysis (see Supplementary Data S1). The aforementioned adipose tissues as well as additional liver and muscle (*M. gluteobiceps femoris*) samples were stored in RNAlater® solution and then used for immunoblotting analysis. We used RNAlater for preservation instead of liquid nitrogen due to the long storage of the samples in field conditions. The use of RNAlater has been previously validated for both RNAlater and liquid nitrogen-preserved samples from western blotting^66,67^.

### RNA extraction, library preparation, and sequencing

RNA extraction, library preparation, and sequencing were performed at The Finnish Functional Genomic Center, Turku, Finland. Total RNA was extracted from adipose tissues (ca <30 mg/sample) using the Qiagen AllPrep DNA/RNA/miRNA Kit according to the manufacturer’s protocol. The quality of the obtained RNA was ensured with an Agilent Bioanalyzer 2100 (Agilent Technologies, Waldbronn, Germany), and the concentration of each sample was measured with a Nanodrop ND-2000 (Thermo Scientific; Wilmington, USA) and a Qbit(R) Fluorometric Quantification, Life Technologies. All samples revealed an RNA integrity number >7.5.

Library preparation was done according to Illumina TruSeq® Stranded mRNA Sample Preparation Guide (part #15031047). Unique Illumina TruSeq indexing adaptors were ligated to each sample to pool several samples later in one flow cell lane. Library quality was inferred with an Advanced Analytical Fragment Analyser and concentration with a Qubit fluorometer, and only good-quality libraries were sequenced.

The samples were normalized and pooled for automated cluster preparation, which was carried out with Illumina cBot station. Libraries prepared for sample YR1_SCAP_322D and FR12_SCAP_402D (Supplementary Data S1) were pooled together and run in one lane to generate a deep sequence to detect lncRNAs for a future study. The remaining 61 libraries were combined in one pool and run on seven lanes of an Illumina HiSeq 3000 platform. Paired-end sequencing with 2 × 75 bp read length was used with a 8 + 8 bp dual index run. Two samples (FR9_SCAP_369D and FR13_PREN_425C) (Supplementary Data S1) were suffering fed pooling error and low amounts of reads and were therefore resequenced in an extra lane. Base calling and adaptor trimming were performed using Illumina’s standard bcl2fastq2 software.

### Bioinformatics analyses

The overall quality of the raw RNA-seq reads in fastq and aligned reads in BAM format were assessed using the FastQC software v0.11.7^68^. FastQC reports were summarized using MultiQC v1.7^69^. High-quality RNA-seq reads for each sample were mapped against the reindeer draft assembly^3^ using Spliced Transcripts Alignment to a Reference (STAR) (version 2.6.0a)^70^ with default parameters. We next generated read counts from the aligned files using the featureCounts software (version 1.6.1) from the Subread package^71^ to assign reads to genes. The GTF-format annotation file associated with the reindeer draft assembly was used for gene coordinate information.

To examine the shared and uniquely expressed genes across the three adipose tissues, we used the cpm function from the edgeR library^72^ to generate CPM values; lowly expressed transcripts with a CPM < 0.5 were discarded.

We hypothesized that there could be differences in seasonal gene expression profiles due to changes in ambient temperature and other climatic factors and subsequent changes in body condition. Hence, we conducted differential gene expression analysis between the spring and winter sampling for each tissue and region using male reindeer samples. Furthermore, to explore regional (and population) differences in gene expression, we compared expression in each tissue between E and F male reindeer. In our study, the analysed group of animals for each tissue included at least three animals from each geographical region and season (Table 1 and Supplementary Data S1).

Raw read counts were processed using the R Bioconductor package DESeq2^73^ to perform differential gene expression and related quality control analysis. Prior to running DESeq2, lowly expressed (rowSums < 1) genes were discarded. Raw gene expression counts were normalized for differences in library size and sequencing depth using DESeq2, to enable gene expression comparisons across samples. We performed PCA to assess sample similarity using the variance stabilizing transformation method. In this study, we used fold-change and false discovery rate (FDR) filtering criteria to identify significantly DEGs. We set absolute value of LFC to be ≥1.5 (|log2FoldChange| > 1.5) and an adjusted *p* value of 0.05 (*p*_adj_ < 0.05) to screen for significant DEGs. The Benjamini–Hochberg FDR method was used to calculate adjusted *p* values.

To gain insight into the biological functions and relevance of the identified DEGs, a functional enrichment analysis was conducted using AgriGO v2.0^74^. In the AgriGO analysis toolkit, to detect the significantly enriched GO terms, default parameters were used in the “Advanced options”: Fisher as the statistical test method, Yekutieli for multiple test correction at a significance level threshold 0.05 (FDR < 0.05), and minimum number of mapping entries 5. In this analysis, the GO annotation file from the de novo assembled reindeer genome^3^ was used as a background reference. Furthermore, to explore the biological pathways associated with the DEGs, we performed KEGG pathway analysis using the GAGE^75^ Bioconductor package. The significantly enriched pathways were identified based on the *q*-values obtained from a Fisher’s exact test (*q*-value < 0.1).

### Immunoblotting analysis of proteins

Adipose tissue samples were homogenized and dissolved in lysis buffer (25 mM Tris [pH 7.4], 0.1 mM EDTA, 1 mM DTT, 15 µl/ml protease inhibitor cocktail (Sigma, St Luis, MO, USA)) to extract total protein content. Insoluble material was removed from the extracts by centrifugation (13,000 × *g*, 10 min, +4 °C). Mitochondrial proteins were extracted from reindeer muscle, liver, reindeer calf prescapular BAT, and mouse BAT samples as described previously^76^. Both total and mitochondrial protein concentrations were determined using the Bradford method (Bio-Rad protein assay, Bio-Rad Laboratories GmbH, München, Germany). Protein extract volumes equivalent to 50–75 μg of total protein were concentrated into smaller volumes by lyophilizing the samples with a Savant Speed Vac Plus SC210A centrifugal evaporator (Thermo Fisher Scientific, Rockford, USA) in cooled conditions for 1 h. Due to the low number of mitochondria in adipose tissue, total protein extractions were used for the immunoblotting of UCP1.

The proteins were separated electrophoretically using a 4–12% gradient gel and transferred to a nitrocellulose membrane (Bio-Rad, Trans-Blot® Transfer Medium, Pure Nitrocellulose Membrane [0.2 μm], Bio-Rad Laboratories, Hercules, CA, USA). The membranes were incubated overnight with UCP1 antibody and loading control protein alpha tubulin antibody for the total protein adipose tissue samples (1:1000 UCP1 Polyclonal Antibody, cat. no. PA1-24894, 1:500 alpha Tubulin Polyclonal Antibody, cat. no. PA5-16891, Invitrogen, Thermo Fisher Scientific, Rockford, USA). Membranes with liver and muscle mitochondrial protein samples were incubated overnight with COX4 antibody (1:1000 COX4 Polyclonal Antibody, cat. no. PA5-17511, Thermo Fisher Scientific, Rockford, USA) and loading control protein ATP5A antibody (1:1000 anti-ATP5A antibody, cat. no. ab151229, Abcam, Cambridge, UK). After the primary antibody treatments, the membranes were incubated with a secondary antibody (1:25,000, Goat anti-Rabbit IgG (H + L) horseradish peroxidase conjugate, cat. no. 31460, Invitrogen, Thermo Fisher Scientific, Rockford, USA) for 1 h. Chemiluminescence for UCP1 and COX4 was detected with SuperSignal West Femto Maximum Sensitivity Substrate (cat. no. 34095, Thermo Fisher Scientific, Rockford, USA) according to the manufacturer’s instructions. Blots were visualized with Odyssey® Fc imaging system (LI-COR Biosciences, Ltd, Cambridge, UK). Positive immunoreactivity for UCP1 with mouse BAT mitochondria and with prescapular BAT mitochondria from newborn reindeer were used as reference samples. Results were normalized with the loading control optical density for alpha tubulin and ATP5A for UCP1 and COX4, respectively.

### Blood metabolites

Plasma leptin concentration was assayed using a multispecies leptin RIA Kit (Cat#XL-85K, Millipore, Billerica, MA, USA). The validity test for reindeer showed a linear correlation between the label and sample concentration. A sensitive bovine, ovine, rat, and mouse insulin RIA kit (Cat#SRI-13K, Millipore, Billerica, MA, USA) was used to measure plasma insulin. Plasma HSL levels were estimated by bovine hormone-sensitive ELISA Kit (Cat#MBS033124, MyBioSource, San Diego, CA, USA). Serum glucose, triglyceride, creatinine, and urea concentrations were determined with enzymatic colorimetric analyses in NordLab, Oulu, Finland.

### Statistical analyses for immunoblotting and blood metabolite analyses

Statistical analysis for the multiple comparisons was performed with an independent-samples Kruskal–Wallis test followed by an independent-samples Mann–Whitney *U*-test. Significant values were adjusted with the Bonferroni correction for multiple tests. Statistical analyses were performed using the IBM SPSS Statistics 21 Data Editor software (IBM, Armonk, NY, USA). *p* Values <0.05 were considered statistically significant. The results of the relative peptide expression levels are presented as mean ± SD.

### Statistics and reproducibility

This study includes RNA-seq of 47 tissue samples from 16 reindeer males that were randomly collected at slaughter from two different geographical regions (Inari, northern Finland and Eveno-Bytantay, Sakha, Yakutia, the Russian Federation) at two seasonal time points: winter (November–December) and spring (April). In physiological analysis, a total of 18 male reindeer were examined, of which 16 were included in the RNA-seq analysis. All bioinformatics and statistical tests were conducted using publicly available programs and packages as described in the “Methods” section above. Reproducibility can be accomplished by following the sample collection conducted in Finland and in the Sakha Republic, the present RNA-sequencing approach, and laboratory methods outlined above.

### Reporting summary

Further information on research design is available in the Nature Research Reporting Summary linked to this article.

## Supplementary information

Supplementary information

Description of Additional Supplementary Files

Reporting Summary

## Data Availability

Raw RNA sequence data analysed in the current study have been deposited in the European Nucleotide Archive (ENA, www.ebi.ac.uk/ena) under study accession number PRJEB44094. Supplementary data files are available online at 10.6084/m9.figshare.15172359.v2 (version 2).
