# Supplementary material for: Adipose gene expression profiles reveal insights into the adaptation of northern Eurasian semi-domestic reindeer (Rangifer tarandus)
Source: Commun Biol. 2021 Oct 7;4:1170. doi: 10.1038/s42003-021-02703-z (PMC8497613; doi:10.1038/s42003-021-02703-z)
Supplement: Supplementary file 1 — Supplementary information [file 42003_2021_2703_MOESM1_ESM.pdf]

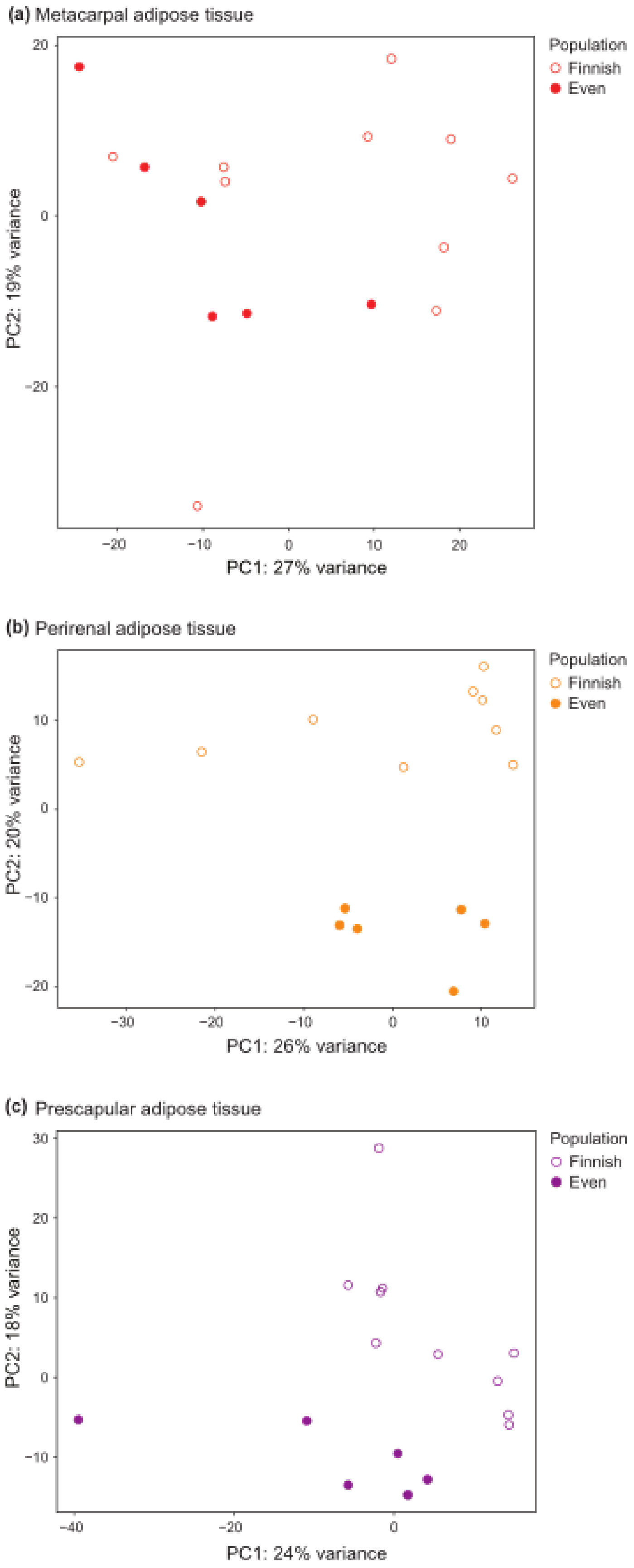

Supplementary Fig. S1 PCA plot based on the region for each tissue. (a) metacarpal, (b) perirenal, and (c) prescapular.

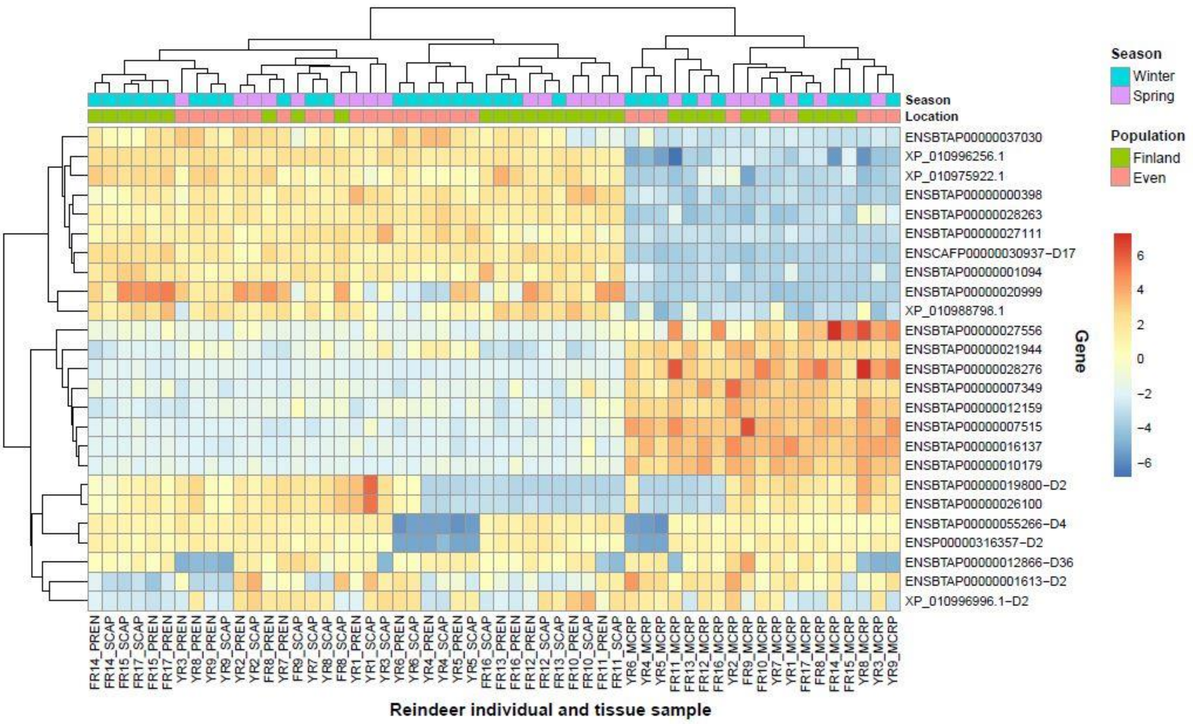

Supplementary Fig. S2 Heatmap plot of the top 25 genes with the highest genetic variance across all samples.

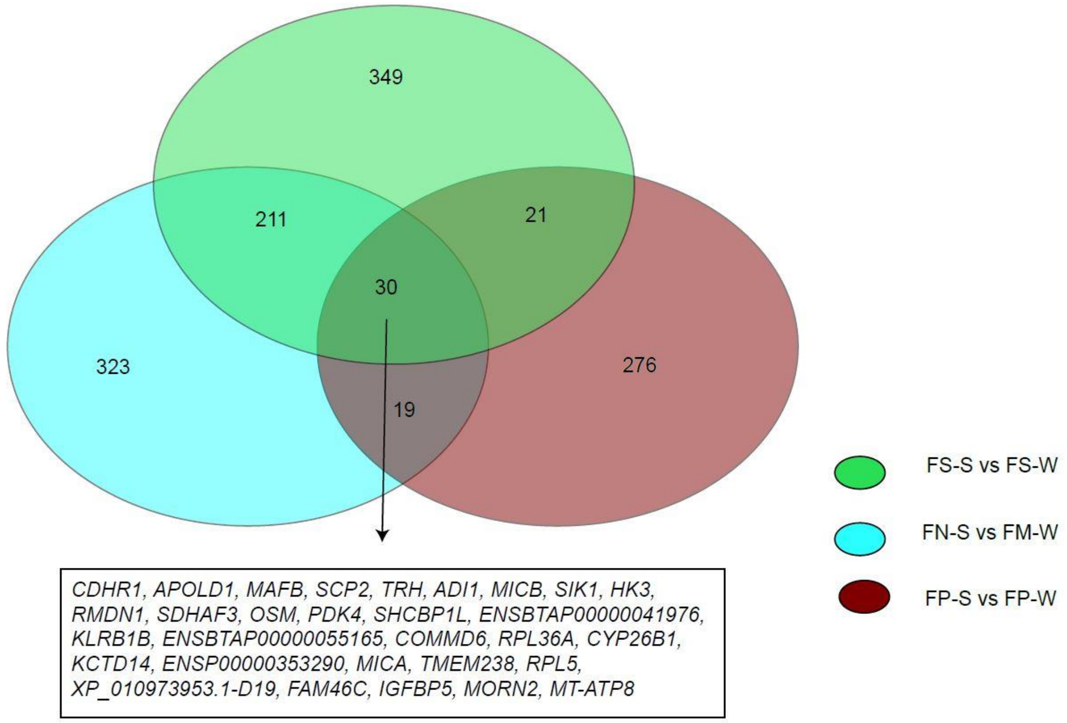

Supplementary Fig. S3 The number of shared and uniquely significant DEGs between three adipose tissues in Finnish reindeer due to seasonal differences. Significant DEGs detected in Finnish reindeer for three adipose tissues due to seasonal change: FM-S vs. FM-W, FP-S vs. FP-W, and FS-S vs. FS-W.

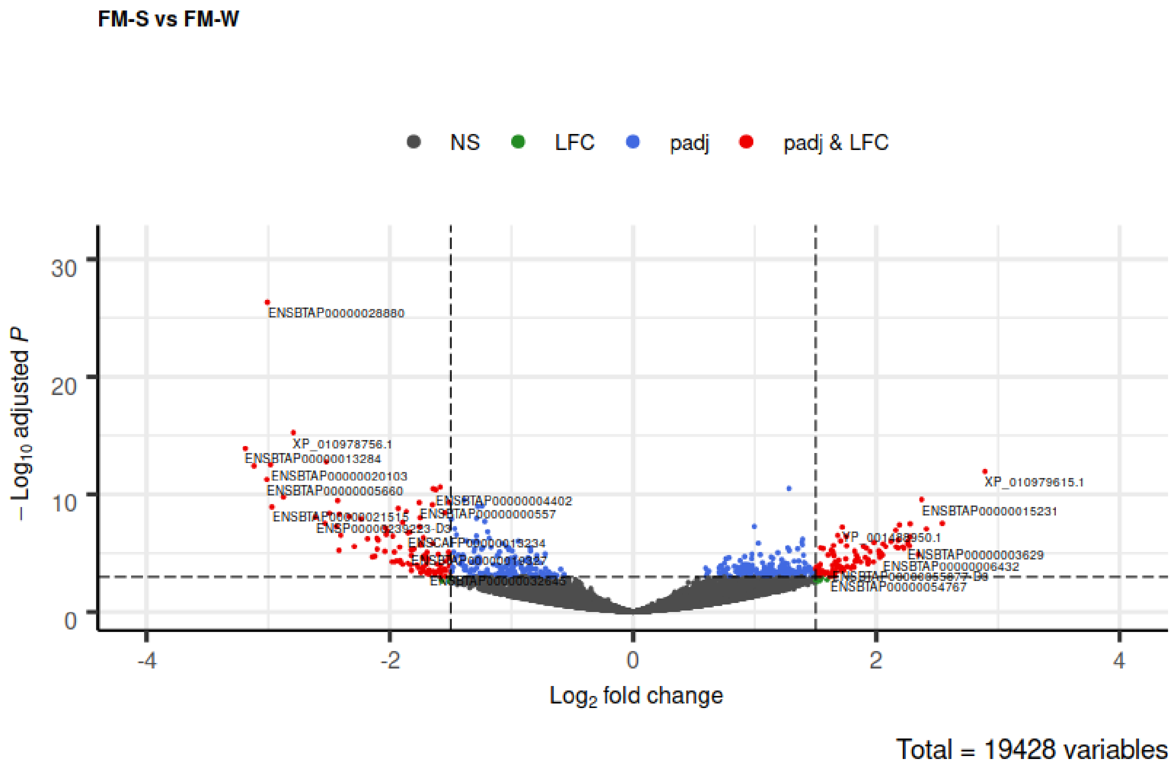

Supplementary Fig. S4 Volcano plot of differentially expressed genes between spring and winter for metacarpal adipose tissue in Finnish reindeer (FM-S vs. FM-W).

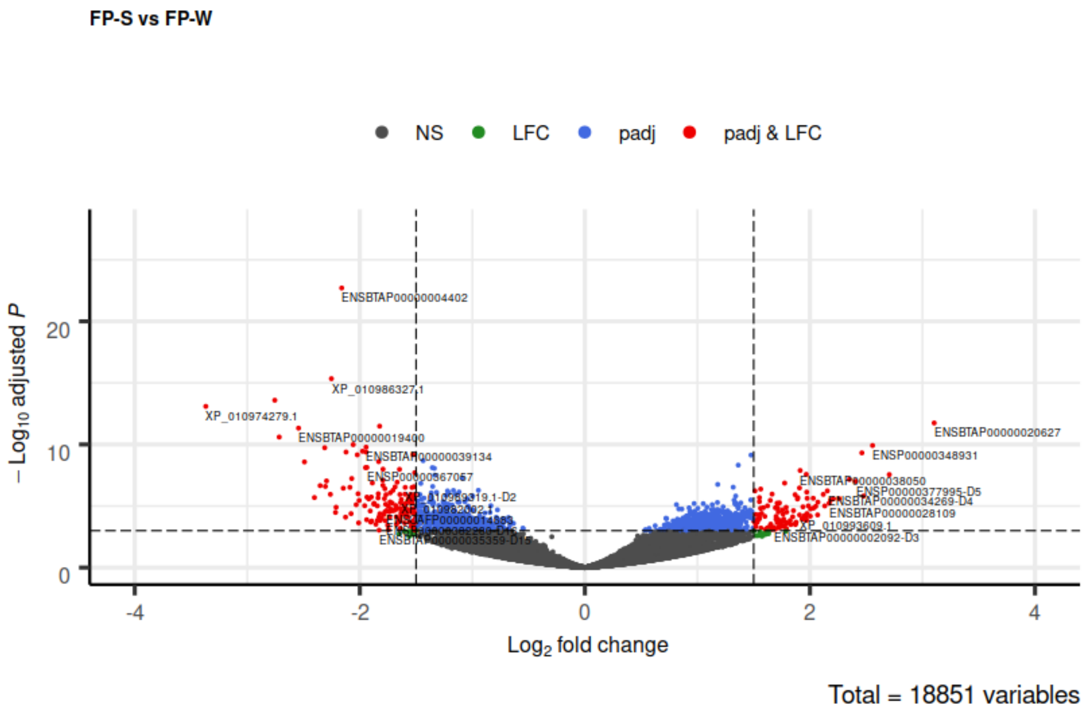

Supplementary Fig. S5 Volcano plot of differentially expressed genes between spring and winter for perirenal adipose tissue in Finnish reindeer (FP-S vs. FP-W).

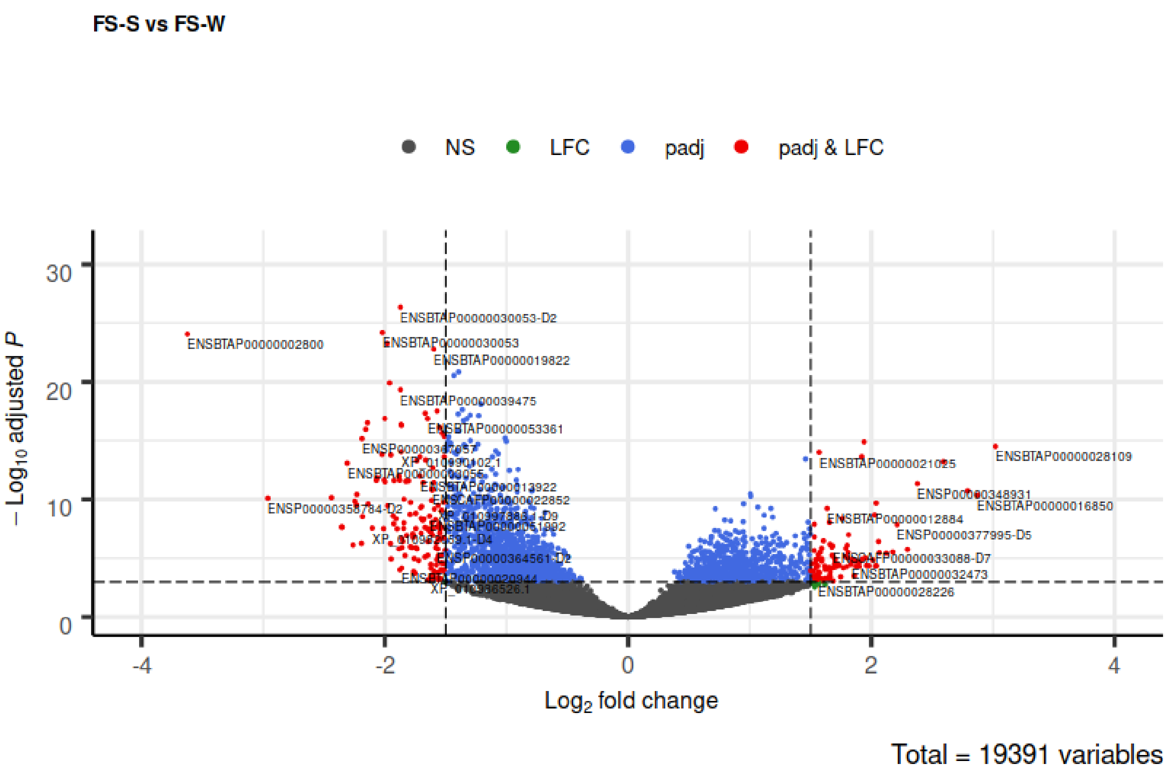

Supplementary Fig. S6 Volcano plot of differentially expressed genes between spring and winter for prescapular adipose tissue in Finnish reindeer (FS-S vs. FS-W).

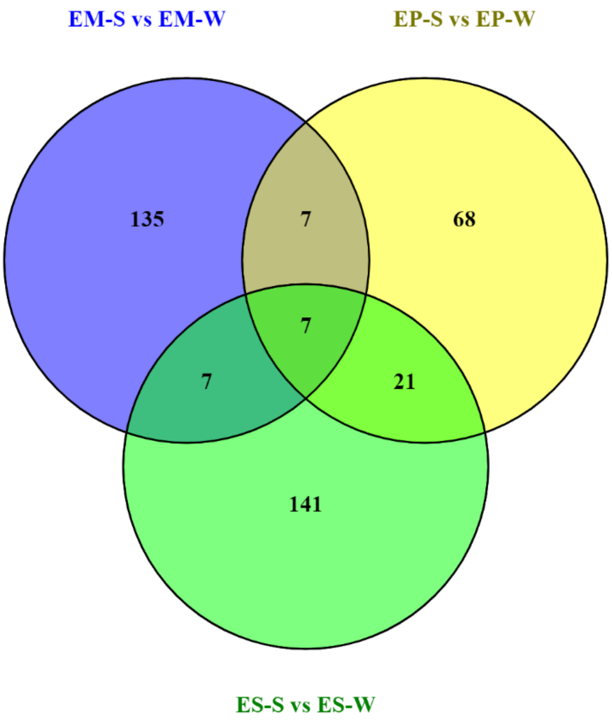

Supplementary Fig. S7 The number of shared and unique significant DEGs between three adipose tissues in Even reindeer due to seasonal differences. Significant DEGs detected in Even reindeer for three adipose tissues due to seasonal change (EM-S vs. EM-W, EP-S vs. EP-W and ES-S vs. ES-W).

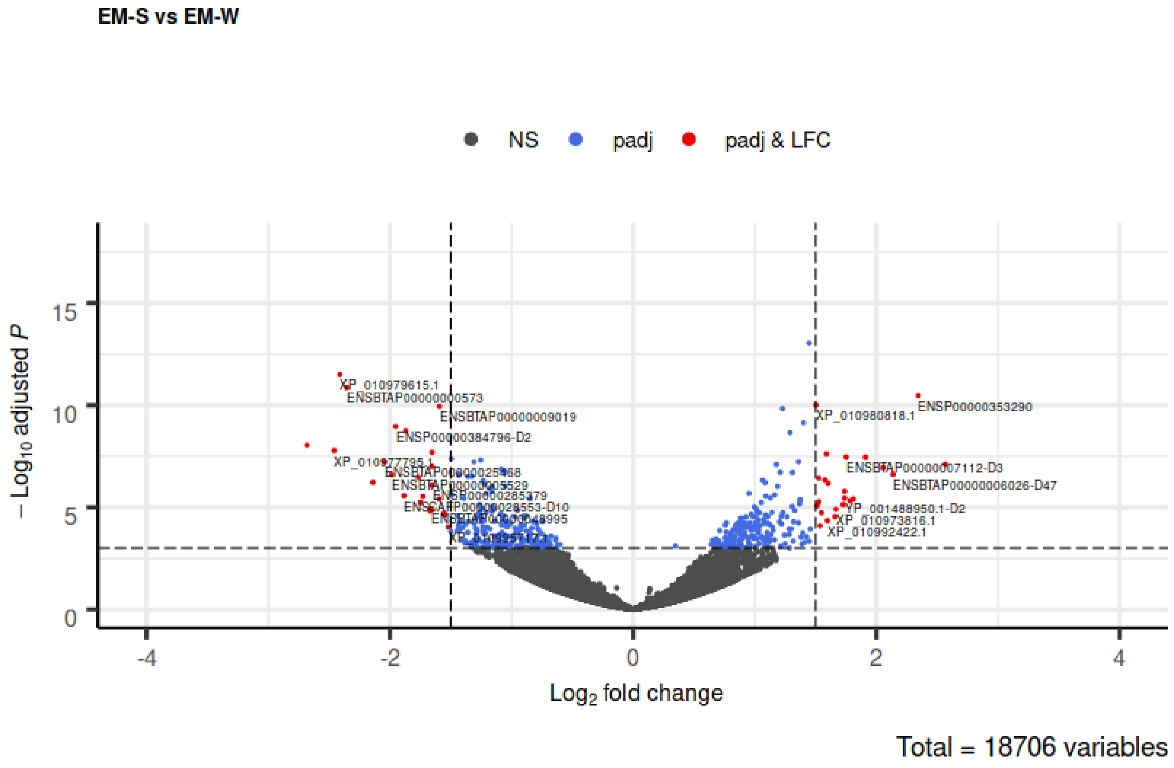

Supplementary Fig. S8 Volcano plot of differentially expressed genes between early spring and early winter for metacarpal adipose tissue in Even reindeer (EM-S vs. EM-W).

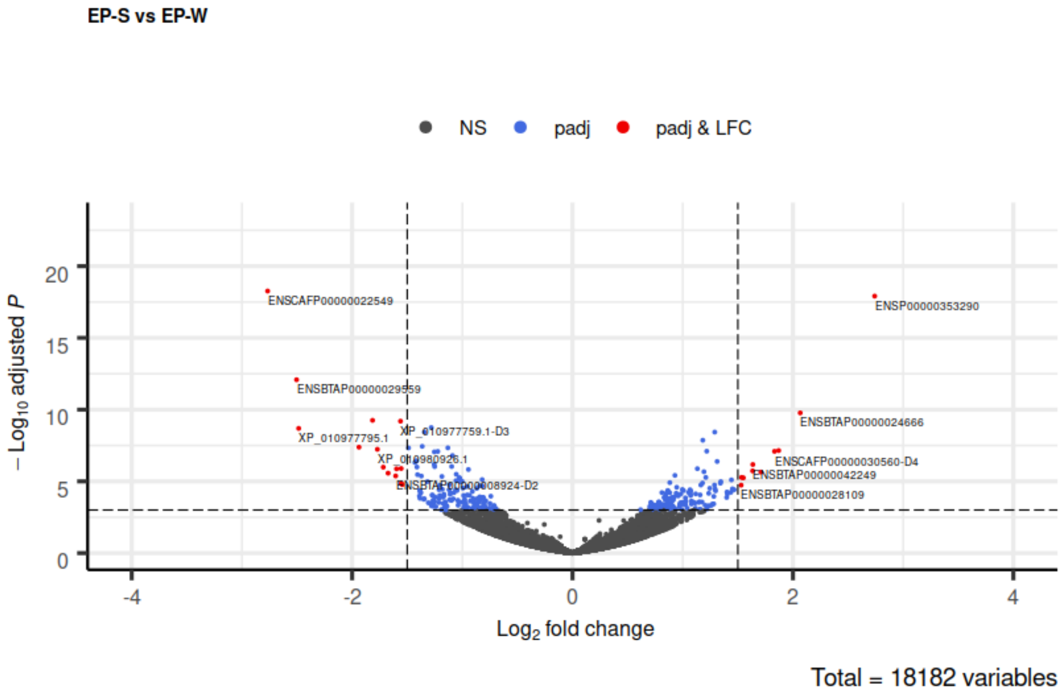

Supplementary Fig. S9 Volcano plot of differentially expressed genes between early spring and early winter for metacarpal adipose tissue in Even reindeer (EM-S vs. EM-W).

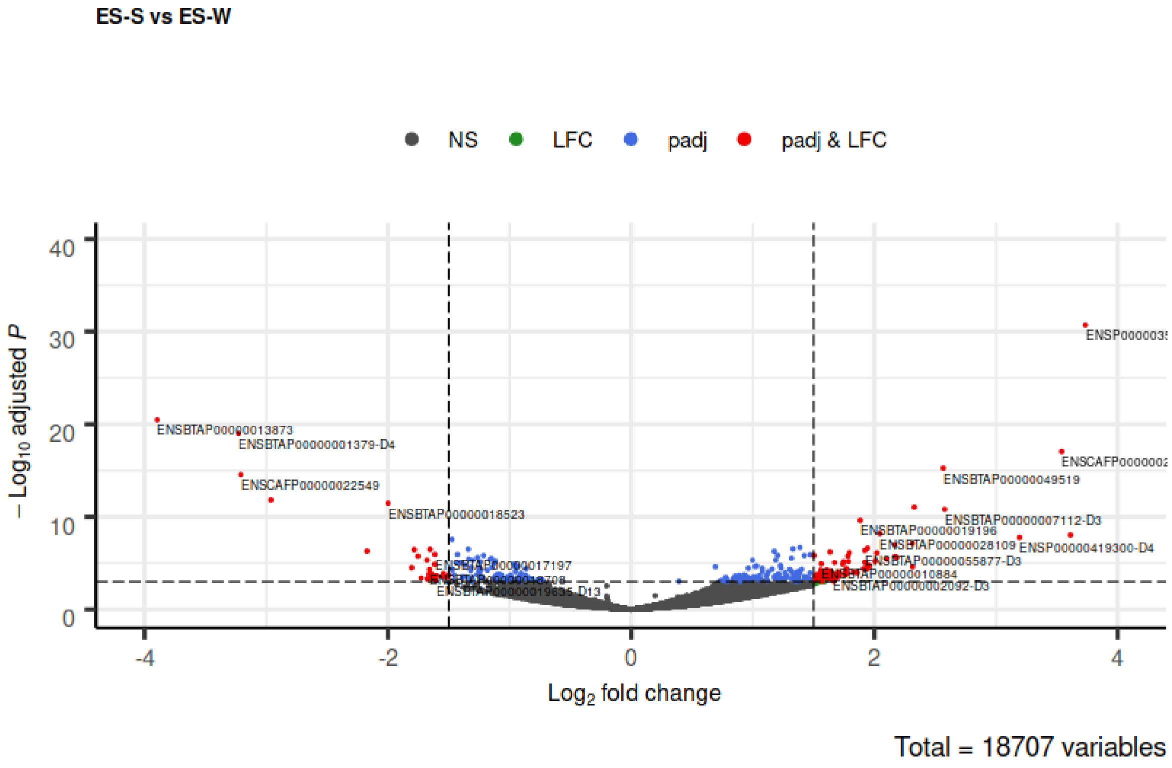

Supplementary Fig. S10 Volcano plot of differentially expressed genes between early spring and early winter for metacarpal adipose tissue in Even reindeer (EM-S vs. EM-W).

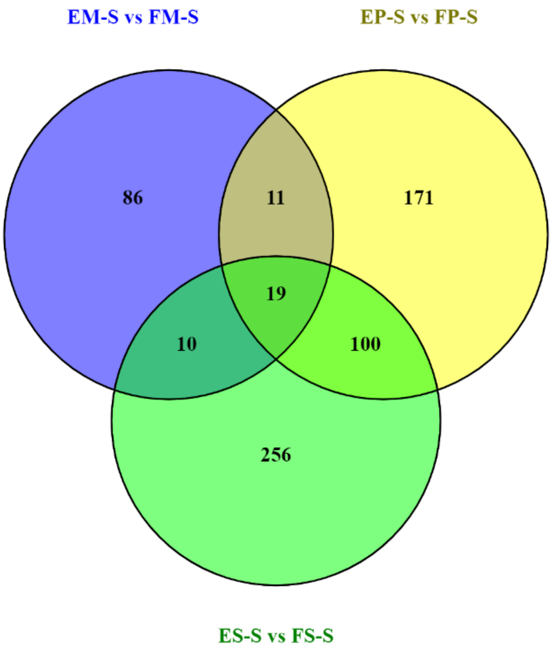

Supplementary Fig. S11 The number of shared and unique significant DEGs in the three adipose tissues detected between Even reindeer and Finnish reindeer due to regional differences in early spring. Significant DEGs detected in three adipose tissues due to regional differences in early spring: EM-S vs. FM-S, EP-S vs. FP-S and ES-S vs. FS-S.

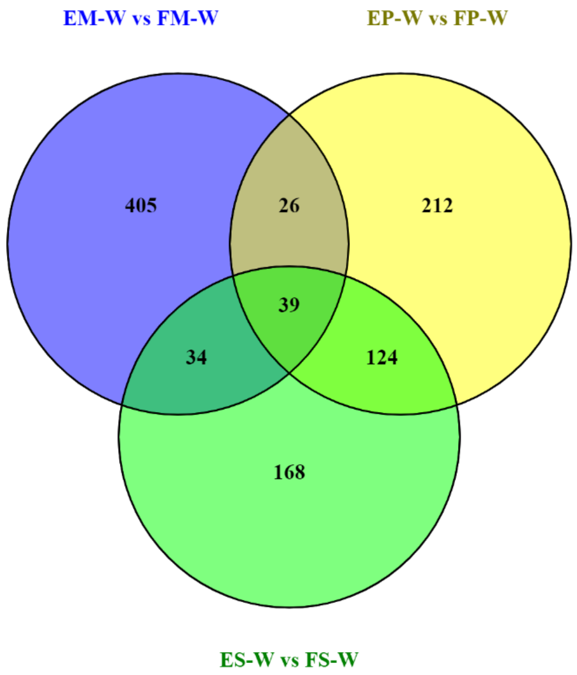

Supplementary Fig. S12 The number of shared and unique significant DEGs in the three adipose tissues detected between Even reindeer and Finnish reindeer due to regional differences in early winter. Significant DEGs detected in three adipose tissues due to regional differences in early winter: EM-W vs. FM-W, EP-W vs. FP-W and ES-W vs. FS-W.

a)

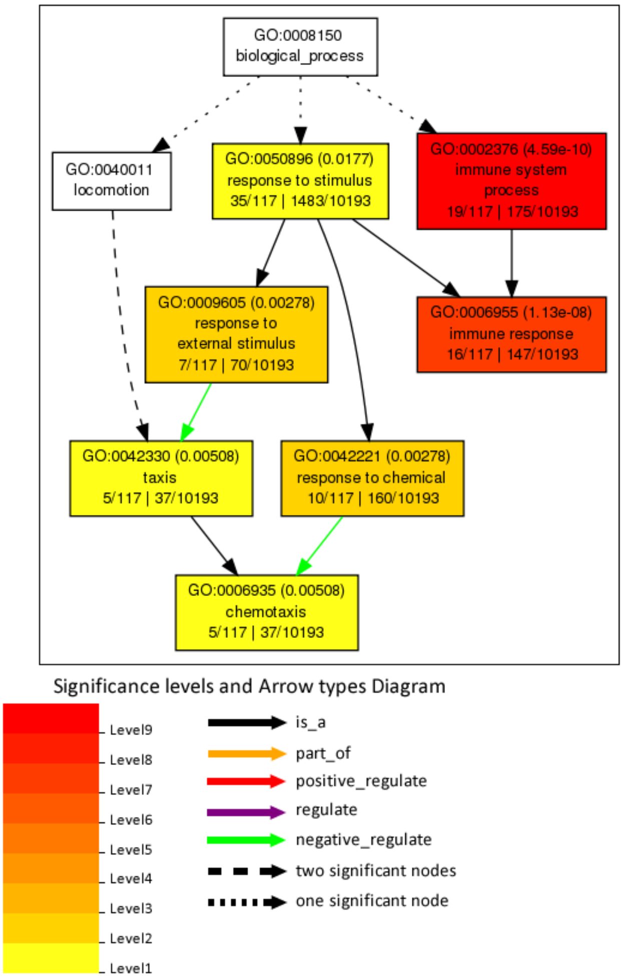

b)

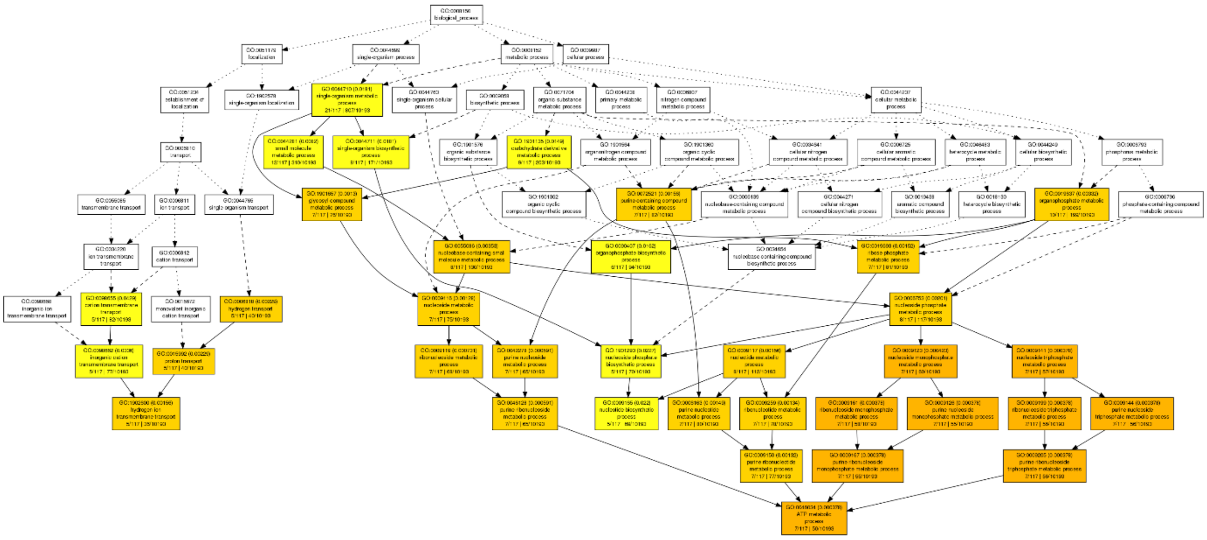

Supplementary Fig. S13. GO hierarchical graph containing all statistically significant GO terms. a) Significantly enriched biological processes GO terms associated with significantly downregulated DEGs in FM-S vs FM-W. b) Significantly enriched biological processes GO terms associated with significantly upregulated DEGs in FM-S vs FM-W.

a)

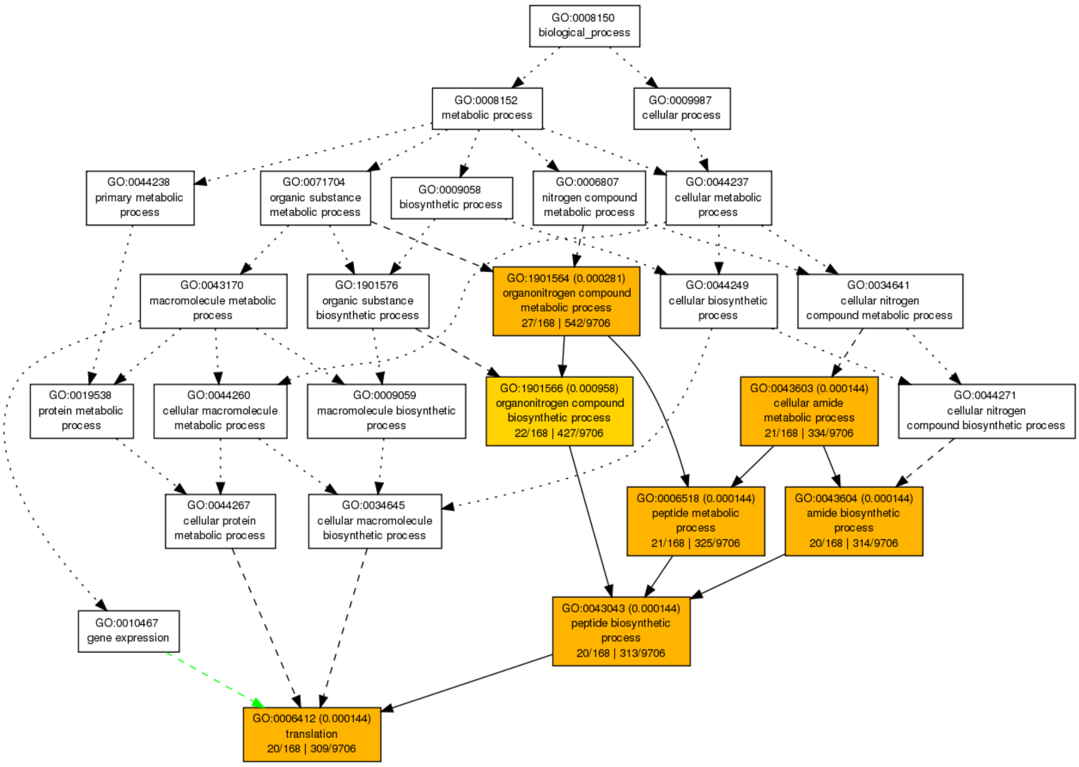

b)

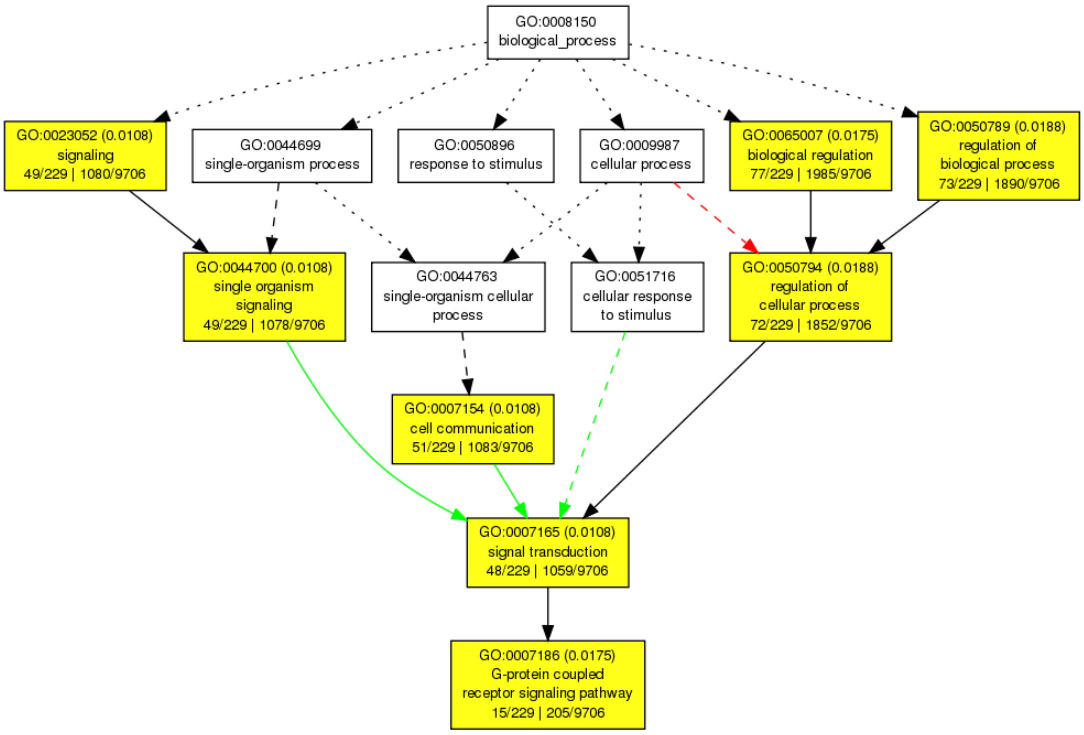

Supplementary Fig. S14. GO hierarchical graph containing all statistically significant GO terms. a) Significantly enriched biological processes GO terms associated with significantly upregulated DEGs in FP-S vs FP-W. b) Significantly enriched biological processes GO terms associated with significantly downregulated DEGs in FP-S vs FP-W.

a)

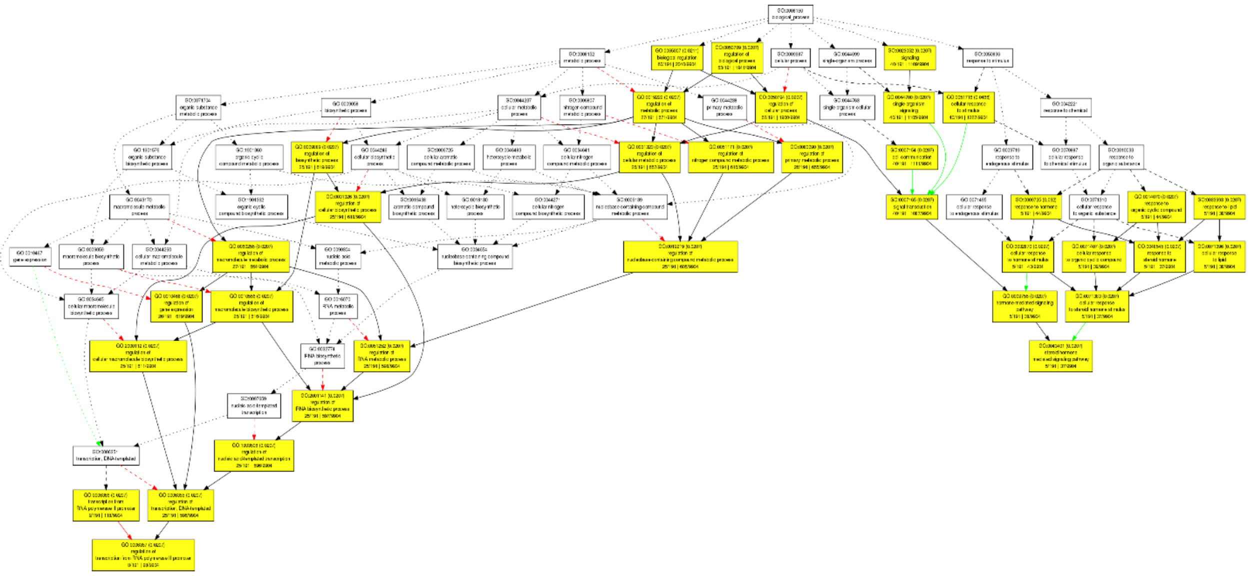

b)

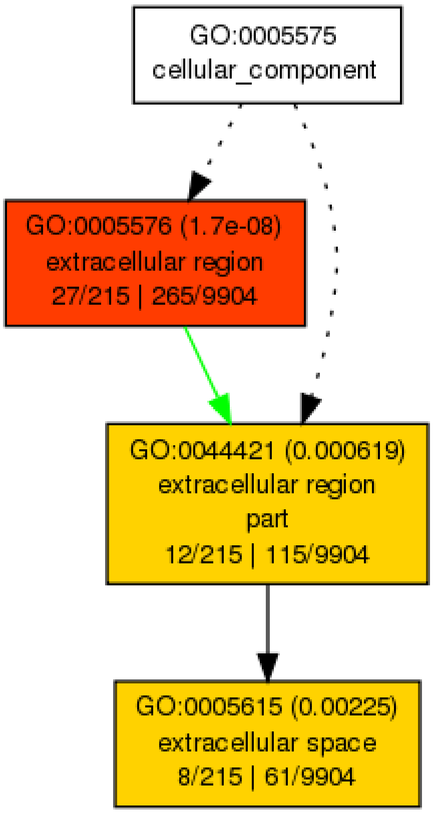

Supplementary Fig. S15. GO hierarchical graph containing all statistically significant GO terms. a) Significantly enriched biological processes GO terms associated with significantly downregulated DEGs in FS-S vs FS-W. b) Significantly enriched cellular component GO terms associated with significantly downregulated DEGs in FS-S vs FS-W.

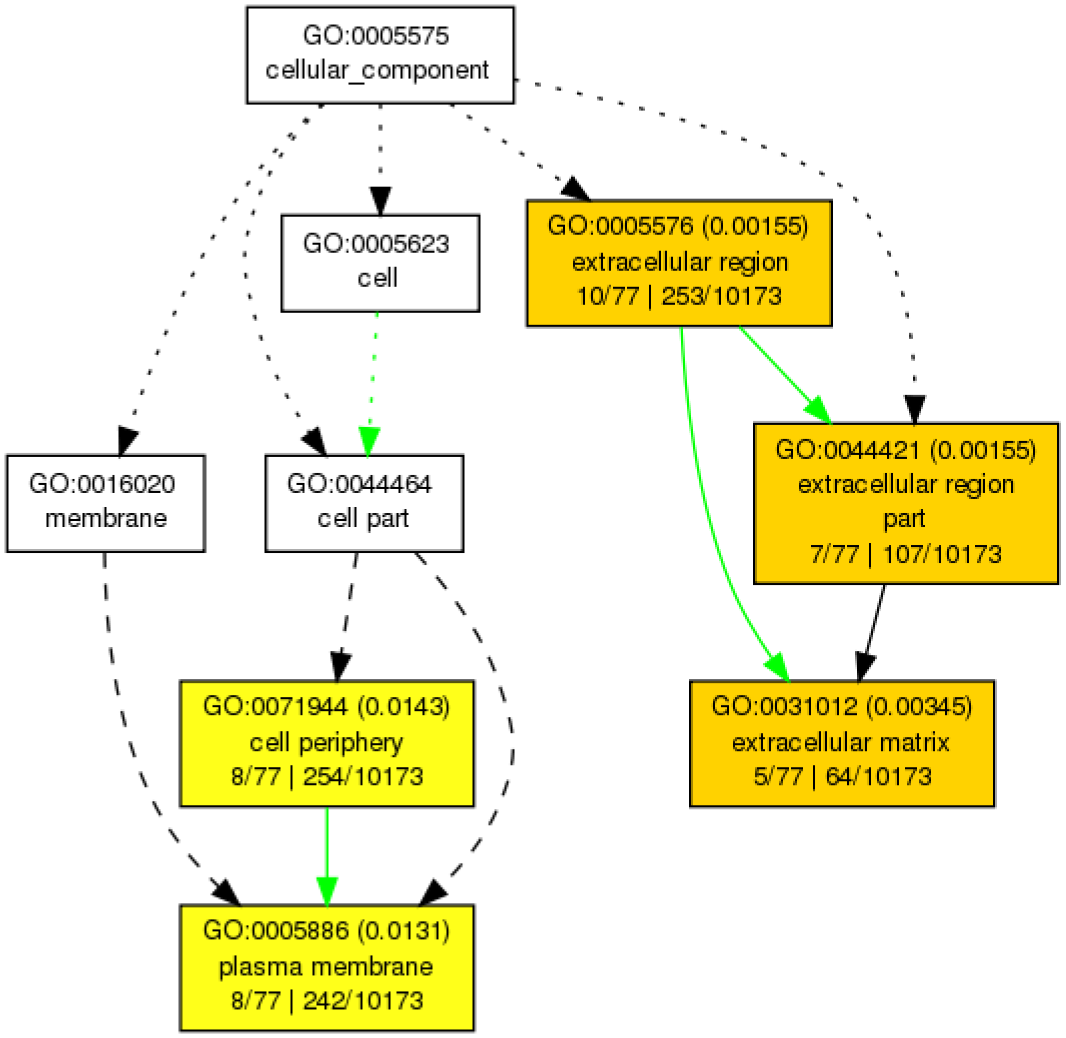

Supplementary Fig. S16. GO hierarchical graph containing all statistically significant GO terms. Significantly enriched cellular component GO terms associated with significantly downregulated DEGs in FM-S vs ES-W.
