# Supplementary material for: Adipose gene expression profiles reveal insights into the adaptation of northern Eurasian semi-domestic reindeer (Rangifer tarandus)
Source: Commun Biol. 2021 Oct 7;4:1170. doi: 10.1038/s42003-021-02703-z (PMC8497613; doi:10.1038/s42003-021-02703-z)
Supplement: Supplementary file 2 — Description of Additional Supplementary Files [file 42003_2021_2703_MOESM2_ESM.pdf]

Description of Additional Supplementary Data Files

Additional Supplementary Data Files are at

https://doi.org/10.6084/m9.figshare.15172359.v2 (version 2).

Supplementary Data S1. Statistics of clean data.

Supplementary Data S2. STAR mapping statistics.

Supplementary Data S3. Summary of expressed genes in each tissue (sheet). List of expressed genes in Finnish reindeer metacarpal adipose tissue in spring (FM-S) (sheet 2). List of expressed genes in Finnish reindeer perirenal adipose tissue in spring (FP-S) (sheet 3). List of expressed genes in Finnish reindeer prescapular adipose tissue in spring (FS-S) (sheet 4). List of expressed genes in Finnish reindeer metacarpal adipose tissue in winter (FM-W) (sheet 5). List of expressed genes in Finnish reindeer perirenal adipose tissue in winter (FP-W) (sheet 6). List of expressed genes in Finnish reindeer prescapular adipose tissue in winter (FS-W) (sheet 7). List of expressed genes in Even reindeer metacarpal adipose tissue in spring (EM-S) (sheet 8). List of expressed genes in Even reindeer perirenal adipose tissue in spring (EP-S) (sheet 9). List of expressed genes in Even reindeer prescapular adipose tissue in spring (ES-S) (sheet 10). List of expressed genes in Even reindeer metacarpal adipose tissue in winter (EM-W) (sheet 11). List of expressed genes in Even reindeer perirenal adipose tissue in winter (EP-W) (sheet 12). List of expressed genes in Even reindeer prescapular adipose tissue in winter (ES-W) (sheet 13).

Supplementary Data S4. List of uniquely expressed genes in the three adipose tissue. List of uniquely expressed genes in metacarpal adipose tissue (sheet 1). List

of uniquely expressed genes in perirenal adipose tissue (sheet 2). List of uniquely expressed genes in prescapular adipose tissue (sheet 3)

Supplementary Data S5. Uniquely expressed genes in metacarpal adipose tissue shared by Finnish and Even reindeer in both seasons.

Supplementary Data S6. Significantly differentially expressed genes between spring and winter in Finnish reindeer metacarpal tissue (FM-S ***vs.*** FM-W).

Supplementary Data S7. Significantly differentially expressed genes between spring and winter in Finnish reindeer perirenal tissue (FP-S ***vs.*** FP-W).

Supplementary Data S8. Significantly differentially expressed genes between spring and winter in Finnish reindeer prescapular tissue (FS-S ***vs.*** FS-W).

Supplementary Data S9. Commonly differentially expressed genes due to seasonal changes in three adipose tissues in Finnish reindeer and Even reindeer.

Supplementary Data S10. Significantly differentially expressed genes between spring and winter in Even reindeer metacarpal tissue (EM-S ***vs.*** EM-W).

Supplementary Data S11. Significantly differentially expressed genes between spring and winter in Even reindeer perirenal tissue (EP-S ***vs***. EP-W).

Supplementary Data S12. Significantly differentially expressed genes between spring and winter in Even reindeer prescapular tissue (ES-S ***vs.*** ES-W).

Supplementary Data S13. Significantly differentially expressed genes between Even and Finnish reindeer in metacarpal adipose tissue in spring (EM-S ***vs.*** FM-S).

Supplementary Data S14. Significantly differentially expressed genes between Even and Finnish reindeer in perirenal adipose tissue in spring (EP-S ***vs.*** FM-S).

Supplementary Data S15. Significantly differentially expressed genes between Even and Finnish reindeer in prescapular adipose tissue in spring (ES-S ***vs.*** FS-S).

Supplementary Data S16. Significantly differentially expressed genes between Even and Finnish reindeer in metacarpal adipose tissue in winter (EM-W ***vs.*** FM-W).

Supplementary Data S17. Significantly differentially expressed genes between Even and Finnish reindeer in perirenal adipose tissue in winter (EP-W ***vs.*** FPW).

Supplementary Data S18. Significantly differentially expressed genes between Even and Finnish reindeer in prescapular adipose tissue in winter (ES-W ***vs.*** FS-W).

Supplementary Data S19. List of significantly enriched GO terms associated with significantly downregulated DEGs (sheet 1) and upregulated DEGs (sheet 2) in Finnish reindeer metacarpal adipose tissue in spring compared to winter.

Supplementary Data S20. List of significantly enriched GO terms associated with significantly downregulated DEGs (sheet 1) and upregulated DEGs (sheet 2) in Finnish reindeer perirenal adipose tissue in spring compared to winter.

Supplementary Data S21. List of significantly enriched GO terms associated with significantly downregulated DEGs (sheet 1) and upregulated DEGs (sheet 2) in Finnish reindeer prescapular adipose tissue in spring compared to winter.

Supplementary Data S22. List of significantly enriched GO terms associated with significantly downregulated DEGs in Even reindeer metacarpal adipose tissue in spring compared to winter.

Supplementary Data S23. List of significantly enriched GO terms associated with significantly downregulated DEGs (sheet 1) and upregulated DEGs (sheet 2) in Even reindeer perirenal adipose tissue compared to Finnish reindeer perirenal adipose tissue in winter.

Supplementary Data S24. List of significantly enriched GO terms associated with significantly

upregulated DEGs in Even reindeer prescapular adipose tissue compared to Finnish

reindeer prescapular adipose tissue in spring.

Supplementary Data S25. List of significantly enriched GO terms associated with significantly downregulated DEGs (sheet 1) and upregulated DEGs (sheet 2) in Even reindeer prescapular adipose tissue compared to Finnish reindeer prescapular adipose tissue in winter.

Supplementary Data S26. List of significantly enriched KEGG pathways associated with significantly downregulated DEGs (sheet 1) and upregulated DEGs (sheet 2) in Finnish reindeer metacarpal adipose tissue in spring compared to winter.

Supplementary Data S27. List of significantly enriched KEGG pathways associated with significantly downregulated DEGs (sheet 1) and upregulated DEGs (sheet 2) in Finnish reindeer perirenal adipose tissue in spring compared to winter.

Supplementary Data S28. List of significantly enriched KEGG pathways associated with significantly downregulated DEGs (sheet 1) and upregulated DEGs (sheet 2) in Finnish reindeer prescapular adipose tissue in spring compared to winter.

Supplementary Data S29. List of significantly enriched KEGG pathways associated with significantly downregulated DEGs in Even reindeer metacarpal adipose tissue compared to Finnish reindeer metacarpal adipose tissue in spring.

Supplementary Data S30. List of significantly enriched KEGG pathway associated with significantly downregulated DEGs in Even reindeer metacarpal adipose tissue compared to Finnish reindeer metacarpal adipose tissue in winter.

Supplementary Data S31. List of significantly enriched KEGG pathways associated with significantly downregulated DEGs (sheet 1) and upregulated DEGs (sheet 2) in Even reindeer perirenal adipose tissue compared to Finnish reindeer perirenal adipose tissue in winter.

Supplementary Data S32. List of significantly enriched KEGG pathways associated with significantly downregulated DEGs (sheet 1) and upregulated DEGs (sheet 2) in

Even reindeer prescapular adipose tissue compared to Finnish reindeer prescapular adipose tissue in winter.

Supplementary Data S33. List of the top 25 most abundant genes expressed in the three adipose tissues based on mean TPM in Finnish reindeer (sheet1) and Even reindeer (sheet2).
