# Supplementary material for: Adipose gene expression profiles reveal insights into the adaptation of northern Eurasian semi-domestic reindeer (Rangifer tarandus)
Source: Commun Biol. 2021 Oct 7;4:1170. doi: 10.1038/s42003-021-02703-z (PMC8497613; doi:10.1038/s42003-021-02703-z)
Supplement: Supplementary file 3 — Reporting Summary [file 42003_2021_2703_MOESM3_ESM.pdf]

nature research

Corresponding author(s): COMMSBIO-21-1017A

Last updated by author(s): Aug 17, 2021

Reporting Summary

Nature Research wishes to improve the reproducibility of the work that we publish. This form provides structure for consistency and transparency in reporting. For further information on Nature Research policies, see our Editorial Policies and the Editorial Policy Checklist.

Statistics

For all statistical analyses, confirm that the following items are present in the figure legend, table legend, main text, or Methods section.

n/a	Confirmed	
☐	☒	The exact sample size (n) for each experimental group/condition, given as a discrete number and unit of measurement
☐	☒	A statement on whether measurements were taken from distinct samples or whether the same sample was measured repeatedly
☐	☒	The statistical test(s) used AND whether they are one- or two-sided<br>*Only common tests should be described solely by name; describe more complex techniques in the Methods section.*
☐	☒	A description of all covariates tested
☐	☒	A description of any assumptions or corrections, such as tests of normality and adjustment for multiple comparisons
☒	☐	A full description of the statistical parameters including central tendency (e.g. means) or other basic estimates (e.g. regression coefficient) AND variation (e.g. standard deviation) or associated estimates of uncertainty (e.g. confidence intervals)
☐	☒	For null hypothesis testing, the test statistic (e.g. F, t, r) with confidence intervals, effect sizes, degrees of freedom and P value noted<br>*Give P values as exact values whenever suitable.*
☒	☐	For Bayesian analysis, information on the choice of priors and Markov chain Monte Carlo settings
☒	☐	For hierarchical and complex designs, identification of the appropriate level for tests and full reporting of outcomes
☒	☐	Estimates of effect sizes (e.g. Cohen's d, Pearson's r), indicating how they were calculated

Our web collection on statistics for biologists contains articles on many of the points above.

Software and code

Policy information about availability of computer code

Data collection	No software was used
Data analysis	(1) Data analysis quality checking: FastQC v0.11.7 and MultiQC v1.7. (2) Alignment: STAR v2.6.0a. (3) Quantification: Featurecounts v1.6.1. (4) Differential gene expression analysis: DESeq2 v1.26.0 (5) GO enrichment analysis: AgriGO v2.0 (6) KEGG pathway analysis: GAGE bioconductor package (7)Immunoblotting and blood metabolite analysis: IBM SPSS Statistics 21 Data Editor software.

For manuscripts utilizing custom algorithms or software that are central to the research but not yet described in published literature, software must be made available to editors and reviewers. We strongly encourage code deposition in a community repository (e.g. GitHub). See the Nature Research guidelines for submitting code & software for further information.

Data

Policy information about availability of data

All manuscripts must include a data availability statement. This statement should provide the following information, where applicable:

- Accession codes, unique identifiers, or web links for publicly available datasets
- A list of figures that have associated raw data
- A description of any restrictions on data availability

Raw sequence reads in compressed fastq format (fastq.gz) analyzed in this study have been deposited to the European Nucleotide Archive (ENA) and are publicly available under project accession PRJEB44094.

Field-specific reporting

Please select the one below that is the best fit for your research. If you are not sure, read the appropriate sections before making your selection.

- [x] Life sciences
- [ ] Behavioural & social sciences
- [ ] Ecological, evolutionary & environmental sciences

For a reference copy of the document with all sections, see nature.com/documents/nr-reporting-summary-flat.pdf

Life sciences study design

All studies must disclose on these points even when the disclosure is negative.

Sample size	The sample sizes were determined based on minimum requirements in cohort comparisons (to obtain "biological power" of the comparsions) in animal genomics and bioinformatics studies. In addition, the possibilities to organize filed work and the availability of animals for sampling effected on the final samples sizes in each cohorts.
Data exclusions	No data were excluded from the analyses.
Replication	The replication of the sampling was not possible because the samples were collected at slaughter (after slaughter) of animals.
Randomization	Not relevant to our study. The allocation into the experimental groups was done based on season, location (population) and gender.
Blinding	Not relevant to our study. The allocation into the experimental groups was done based on season, location (population) and gender. The aim of the study was to investgated gene expression differences between these groups.

Reporting for specific materials, systems and methods

We require information from authors about some types of materials, experimental systems and methods used in many studies. Here, indicate whether each material, system or method listed is relevant to your study. If you are not sure if a list item applies to your research, read the appropriate section before selecting a response.

Materials & experimental systems

n/a	Involved in the study	
x		Antibodies
x		Eukaryotic cell lines
x		Palaeontology and archaeology
	x	Animals and other organisms
x		Human research participants
x		Clinical data
x		Dual use research of concern

Methods

n/a	Involved in the study	
x		ChIP-seq
x		Flow cytometry
x		MRI-based neuroimaging

Animals and other organisms

Policy information about studies involving animals; ARRIVE guidelines recommended for reporting animal research

Laboratory animals	Laboratory animals were not used in the study.
Wild animals	In the present study, tissue samples of semi-domesticated reindeer (Rangifer tarandus) animals were collected at slaughter. These animals were not wild ones. The animals were slaughtered for meat production.
Field-collected samples	The Finnish samples were collected at a reindeer slaughter house, while the Sakha samples were collected outside (the slaughtering of reindeer typilcally occurs outisde there). The mean daily temperature in Inari, Finland varied between -16.1C and 5.2C before the sampling in winter (14 hours light, 10 hours dark) and between -13.2C and +4C before the sampling in spring (16 hours light, 8 hours dark). In northern Sakha, the daily temperature varied between -13C and -24C during the winter sampling (6.5 hours daylight, 17.5 hours dark) and between -9C and - 0C during the spring sampling (14 hours of daylight, 10 hours dark).
Ethics oversight	All protocols and sample collections were performed in accordance with the legislations approved by the Russian authorization board (FS/UVN 03/163733/07.04.2016) and the Animal Experiment Board in Finland (ESAVI/7034/04.10.07.2015).

Note that full information on the approval of the study protocol must also be provided in the manuscript.
